# Pharmacological effects of dihydromyricetin in preclinical models of NAFLD: a systematic review and meta-analysis stratified by dose and duration

**DOI:** 10.3389/fphar.2026.1863189

**Published:** 2026-08-06

**Authors:** Le Li, Jiahui Chen, Wenxin Hu, Jiachen Wang, Fangran Wang, Chenxuan Dong, Lisha Wang, Tianying Chang, Jiajuan Guo, Yingzi Cui, Xing Liao

**Affiliations:** 1 College of Traditional Chinese Medicine, Changchun University of Chinese Medicine, Changchun, China; 2 GCP Department, The Affiliated Hospital of Changchun University of Chinese Medicine, Changchun, China; 3 Administration Department, The Affiliated Hospital of Changchun University of Chinese Medicine, Changchun, China; 4 Office of the Dean of Academic Affairs, Changchun University of Chinese Medicine, Changchun, China; 5 Center for Evidence-based Chinese Medicine, Institute of Basic Research in Clinical Medicine, China Academy of Chinese Medical Sciences, Beijing, China

**Keywords:** animal model, dihydromyricetin, dose and duration, meta-analysis, non-alcoholic fatty liver disease, systematic review

## Abstract

**Background:**

Dihydromyricetin (DHM), a naturally occurring flavonoid, has shown beneficial effects in preclinical models of non-alcoholic fatty liver disease (NAFLD). Nevertheless, the extent to which dose and treatment duration influence its pharmacological effects remains to be systematically evaluated.

**Objective:**

This study aimed to evaluate the pharmacological effects of DHM in preclinical models of NAFLD and to further explore the potential influence of treatment dose and duration on these effects using subgroup analyses.

**Methods:**

Studies were identified through systematic searches of six electronic databases. Eligible animal studies were included according to predefined criteria. Risk of bias (RoB) was assessed using the SYRCLE tool. Quantitative synthesis was performed using *RevMan* (version 5.4). To further explore the potential influence of treatment dose and duration, three-dimensional (3D) visualization plots and radar charts were generated using *OriginPro 2024*.

**Results:**

A total of 13 studies encompassing 14 independent experiments with 316 animals were included. DHM was associated with improvements in hepatic and serum lipid profiles, liver enzyme levels, and glucose homeostasis. Heterogeneity remained high across strata despite subgroup analyses by dose and duration. Integrated 3D plots and radar charts visually illustrated these findings.

**Conclusion:**

DHM exhibited favorable pharmacological effects in preclinical models of NAFLD, with lipid-related outcomes appearing more sensitive to dose and liver enzyme outcomes showing greater responsiveness to treatment duration. Persistent heterogeneity highlights the need for standardized experimental protocols in future preclinical research.

**Systematic Review Registration:**

https://www.crd.york.ac.uk/PROSPERO/view/CRD420251124831, identifier CRD420251124831.

## Introduction

Non-alcoholic fatty liver disease (NAFLD) is the most prevalent chronic liver disease globally (Tilg et al., 2023; Targher et al., 2021). Currently, approximately 38% of the global population is affected by NAFLD (Adinolfi et al., 2026; Wong et al., 2023; Younossi et al., 2025), with a higher prevalence in men than women (Teng et al., 2023). The incidence and burden of NAFLD have been steadily increasing (Paik et al., 2023). NAFLD is a major cause of end-stage liver disease (Lin et al., 2026; Shang et al., 2026). It is closely associated with the development of hepatocellular carcinoma (Kanwal et al., 2018; Fujiwara et al., 2022; Wong et al., 2022). Furthermore, it is linked to an elevated risk of extra-hepatic complications (Kim et al., 2024; Huang et al., 2022; Duan et al., 2023; Byrne and Targher, 2020; Lonardo et al., 2018), including type 2 diabetes, cardiovascular disease (Yan et al., 2026), and chronic kidney disease, which are the leading causes of death in affected individuals (Li et al., 2023; Liu et al., 2025). Despite the role of lifestyle interventions in managing NAFLD (Montemayor et al., 2023), there are currently no FDA-approved pharmacological treatments (Sanyal, 2019; Baghdadi et al., 2025), and existing therapeutic options offer limited and often insufficient long-term efficacy (Katsiki et al., 2016; Boushehri et al., 2025). Pharmacological interventions mainly include insulin sensitizers, lipid-lowering agents, and antioxidants. Although these agents show certain benefits in reducing hepatic steatosis and inflammation, their long-term use is often limited by adverse effects, inconsistent efficacy, high costs, and poor patient adherence (Lombardi et al., 2017). Consequently, there is an urgent need to develop novel therapeutic agents to address this unmet clinical need and improve outcomes for patients with NAFLD. As emphasized in recent discussions on evidence-based approaches to traditional medicine, rigorous synthesis of existing data is essential for translating empirical observations into actionable clinical guidance (Liu and Robinson, 2025).

Natural products constitute a valuable reservoir for drug discovery, particularly in the field of metabolic diseases (Atanasov et al., 2021; Jiang et al., 2025; Karimi et al., 2025). Numerous bioactive plant metabolites have provided crucial scaffolds for the development of novel therapeutics (Harvey et al., 2015; Yang et al., 2024a). Among them, dihydromyricetin (DHM), a flavonoid isolated from Ampelopsis grossedentata and related species, has gained considerable attention (Wang et al., 2023). Ampelopsis grossedentata, commonly known as vine tea, has been consumed for centuries in southern China as a traditional botanical drug and functional food (Wu et al., 2023). DHM is recognized as the predominant flavonoid metabolite of this plant (Liu et al., 2019; Wang et al., 2025c; Fan et al., 2017), providing a well-defined phytochemical basis for metabolite standardization and pharmacological investigation. In traditional medicine, vine tea has been used to relieve fatigue, hangovers, and liver-related disorders (Zhang et al., 2021; Qi et al., 2024b). Similar applications have been documented in Japan and Southeast Asia, where extracts of Ampelopsis species are incorporated into botanical remedies and functional foods for their antioxidant and hepatoprotective effects (Luo et al., 2023). Traditionally consumed as a medicinal tea for liver-related conditions and general health promotion, DHM has recently been intensively studied for its pharmacological potential (Chen et al., 2021; Zhang et al., 2025; He et al., 2024; Tan et al., 2022). These long-standing applications provide an important ethnopharmacological rationale for the further pharmacological development of DHM. Increasing evidence demonstrates that DHM possesses a wide range of bioactivities, including antioxidant, anti-inflammatory, lipid-lowering, and insulin-sensitizing effects (Wang et al., 2025c; Yang et al., 2019), underscoring its potential in the treatment of metabolic disorders such as NAFLD (Wang et al., 2022; Zhang et al., 2018a; Chen et al., 2015; Niazpour and Meshkani, 2025).

Mechanistically, DHM regulates multiple signaling pathways implicated in NAFLD pathogenesis (Gong et al., 2022; Tong et al., 2020). It activates AMP-activated protein kinase (AMPK) while suppressing sterol regulatory element-binding protein-1c (SREBP-1c) and downstream lipogenic enzymes, thereby improving hepatic lipid metabolism and reducing lipid accumulation (Xie et al., 2016; Zhou et al., 2017). Moreover, DHM mitigates oxidative stress by strengthening endogenous antioxidant defenses and attenuates hepatic inflammation by inhibiting NF-κB and NLRP3 inflammasome activation, ultimately preserving hepatocellular function (Sun et al., 2021). Emerging studies further reveal that DHM modulates gut microbiota composition and restores intestinal barrier integrity, collectively reducing endotoxin-driven hepatic inflammation and metabolic disturbance (Kang et al., 2024; Porras et al., 2017). These pleiotropic effects contribute to the restoration of hepatic homeostasis (Le et al., 2016). Given its multitarget pharmacological profile, DHM is suited to address the complex pathophysiology of NAFLD. Accumulating preclinical evidence suggests that DHM exerts beneficial pharmacological effects on hepatic steatosis, inflammation, and fibrosis in experimental NAFLD models (Zhou et al., 2021b), supporting its further development for NAFLD.

However, the available evidence varies considerably across studies in terms of experimental model, treatment dose, and outcome assessment. Whether dose or treatment duration influences the pharmacological effects of DHM remains unclear. This study further explored these factors through stratified meta-analyses and descriptive dose- and duration-based visualizations in preclinical models of NAFLD.

## Methods

This study has been registered in PROSPERO with the registration number CRD 420251124831. The study was conducted in accordance with the Preferred Reporting Items for Systematic Reviews and Meta-Analyses (PRISMA) statement (Page et al., 2021).

### Search strategy

We systematically searched PubMed, Embase, Web of Science, and the Cochrane Library, as well as Chinese databases including China National Knowledge Infrastructure (CNKI), Wanfang Database, and China Science and Technology Citation Database (VIP), from database inception to August 2025. The search strategy combined Medical Subject Headings (MeSH) or EMTREE terms with free-text keywords related to NAFLD and DHM. Searches were limited to animal experimental studies published in English or Chinese. In addition, the reference lists of included articles were manually screened to identify potentially relevant studies. The detailed search strategies for each database are provided in Supplementary Material 1.

### Inclusion and exclusion criteria

The inclusion criteria were determined using the Population, Intervention, Control, and Outcome (PICO) principle.

We adopted the following predefined inclusion criteria: (1) The study subjects are experimental animal models of NAFLD, with no restrictions on the methods of model creation or animal species; (2) The study design is a randomized controlled trial; (3) The intervention is the treatment group receiving DHM alone, with no restrictions on the administration route or dose form. The model group, after successful modeling, receives an equal volume of physiological saline or distilled water as the control or blank control, with no limitations on the starting time or duration of the experiment; (4) The outcome measures include body weight, liver-to-body weight ratio (%), total cholesterol (TC) levels in liver tissue, triglyceride (TG) levels in liver tissue, serum TC, serum TG, high-density lipoprotein (HDL-C), low-density lipoprotein (LDL-C), alanine aminotransferase (ALT), aspartate aminotransferase (AST), fasting blood glucose and fasting insulin levels.

Studies were excluded if they involved clinical participants or *in vitro* experiments, review articles, conference abstracts, studies lacking extractable data, investigations employing combined interventions, or duplicate publications; in the latter case, the most recent report was retained.

### Data extraction

Two investigators (LL and CTY) independently extracted relevant data using a standardized form. Extracted information included bibliographic details, experimental design, animal characteristics, modeling procedures, intervention protocols, and outcome measures. When outcomes were reported at multiple time points, data corresponding to the end of the intervention period were preferentially collected. For studies presenting results only in graphical form, numerical values were retrieved using *GetData Graph Digitizer* (version 2.26) (Zhou et al., 2023; Zhou et al., 2021a; Xu et al., 2025). In experiments with multiple treatment arms sharing a common control group, control data were proportionally allocated to avoid unit-of-analysis errors. Discrepancies between reviewers were resolved through discussion or consultation with a third investigator (GJJ).

### Risk bias evaluation

Methodological rigor was evaluated using the SYRCLE RoB tool for animal studies (Hooijmans et al., 2014). This tool assesses the following domains: sequence generation, baseline comparability, allocation concealment, housing conditions, blinding procedures, outcome assessment, data completeness, selective reporting, and other potential sources of bias. Each domain was categorized as low, high, or unclear risk. Quality assessment was independently performed by two reviewers (LL and CTY), with disagreements adjudicated by a third reviewer (CYZ).

### Statistical analysis

Meta-analyses were conducted using *RevMan* (version 5.4). Continuous variables were summarized as mean differences (MD) or standardized mean differences (SMD), with corresponding 95% confidence intervals (CI), depending on the consistency of measurements across studies. Statistical significance was defined as *P* < 0.05. Between-study heterogeneity was evaluated using the I^2^ statistic. A random-effects model was applied when substantial heterogeneity was detected (I^2^ > 50%); otherwise, a fixed-effects model was used. Sensitivity analyses were conducted to assess the robustness of the pooled estimates. Due to the limited number of rat studies and considerable methodological heterogeneity, a quantitative synthesis was not performed; instead, the studies were described narratively. All subsequent subgroup analyses were restricted to mouse studies. We conducted subgroup analyses in *RevMan* by experimental model, dose, and treatment duration to assess whether the pharmacological effects of DHM differed across study conditions and to further explore the influence of dose and treatment duration on key metabolic and hepatic outcomes. We further stratified the included studies according to the main pathophysiological features of each experimental model. After data extraction was completed, dose and treatment duration thresholds were determined based on the distribution of studies included in the analysis. Studies were stratified by dose into low-dose (<100 mg/kg/day) and high-dose (≥100 mg/kg/day) regimens, and by treatment duration into short-term (≤8 weeks) and long-term (>8 weeks) interventions. Publication bias was assessed using a funnel plot when at least ten studies were available. To further explore dose-response and duration-effect relationships, three-dimensional (3D) plots and radar charts were generated using *OriginPro* 2024.

## Results

### Study selection

**FIGURE 1:**
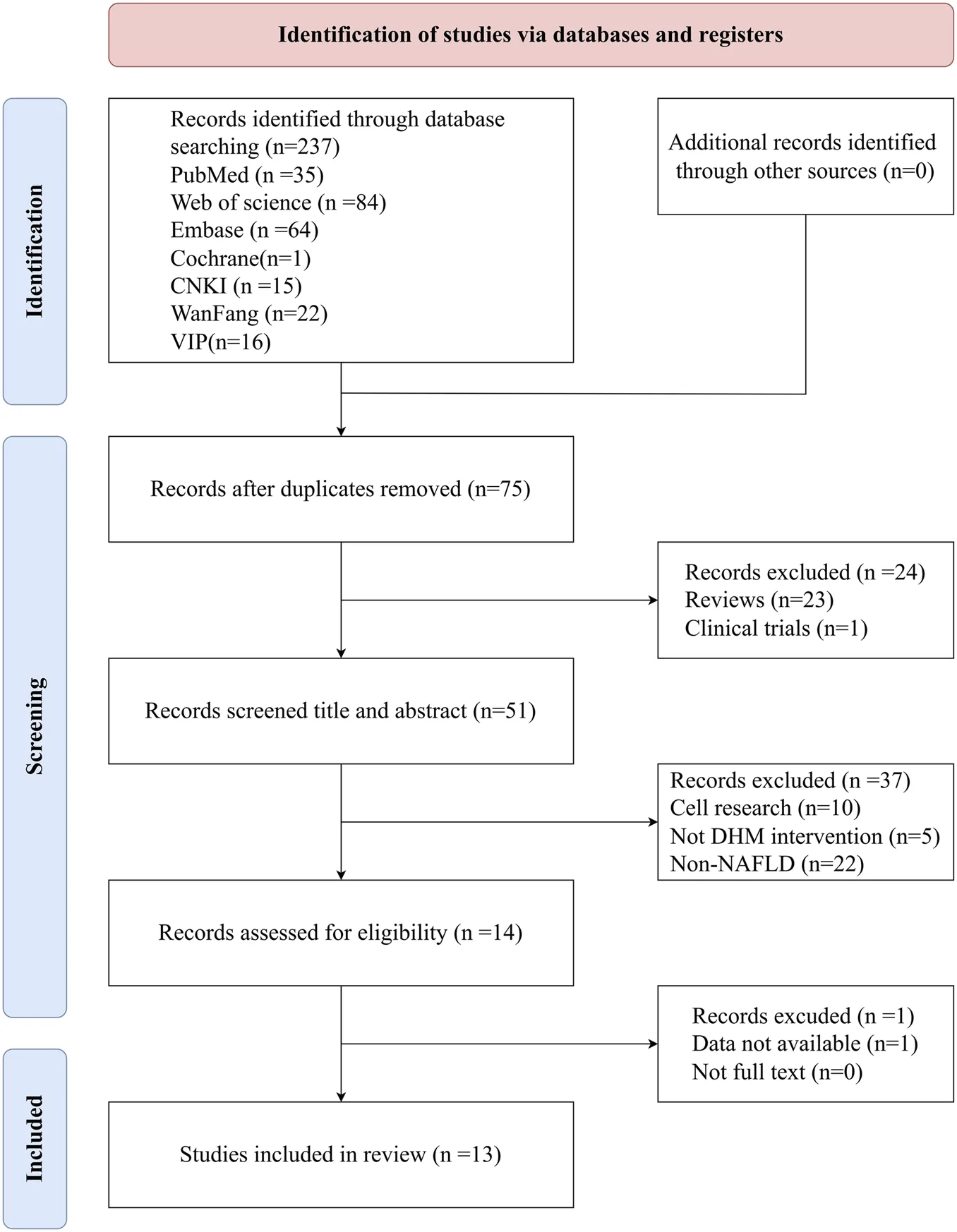
Flow chart of study selection.

According to the search strategy described above, 237 articles published from the establishment of each database to August 2025 were retrieved from seven databases. The process of literature screening is illustrated in Figure 1. First, 162 duplicate records were excluded. Second, 61 articles were removed, including reviews, irrelevant studies, master’s or doctoral dissertations, articles not published in core Chinese journals, and studies involving combination therapies. Following a thorough examination of the extant literature, one study (Liu et al., 2023b) was excluded due to an unspecified sample size. In conclusion, a total of 13 studies (Guo et al., 2019; Kang et al., 2024; Li et al., 2024a; Liu, 2017; Liu et al., 2023a; Ma et al., 2018a; Ma et al., 2018b; Miao et al., 2023; Song et al., 2022; Yang et al., 2024c; Zeng et al., 2019; Zeng et al., 2020; Zhu et al., 2018) were selected for inclusion in the analysis, with the studies being conducted in both Chinese and English languages.

### Characteristics of included studies

Thirteen studies reported a total of fourteen independent experiments, with one study (Zeng et al., 2019) reporting two independent experiments (each experimental group was counted separately, and the number of experiments was denoted as “n”). Detailed information is presented in Table 1.

**TABLE 1:** Characteristics of included studies.

Study, year	Species	Sex	Age	Sample size (n) treatment/Control	Initial body weight	Model	Composition of the high-fat diet	Purity of DHM	Source of DHM	Intervention		Anesthetic	Outcome	Statistical different
										Treatment	Control			
Guo et al. (2019)	SD rat	Male	4–5 weeks	7/7	170 ± 20 g	HFD feeding for 12 weeks	Containing 36.5% fat, 44.6% carbohydrates, and 18.9% protein	NR	Purchased from Sigma-Aldrich; Merck KGaA, Darmstadt, (Germany)	Intravenous injection of DHM (2 mg/kg) every 3 days	Control group (no treatment)	Anesthetized with sodium pentobarbital (35 mg/kg, intraperitoneally).	1, 6, 9, 10	1. p < 0.01 6. p < 0.01 9. p < 0.01 10. p < 0.01
Kang et al. (2024)	C57BL/6 mice	Male	3–4 weeks	10/10	16–18 g	HFD feeding for 4 weeks	Commercial standard feed (Model DH10045) with a fat-to-energy ratio of 45%	98%	Purchased from Shanghai Biotechnology Co., Ltd., (China)	Oral gavage of DHM (1000 mg/kg) for 8 weeks	Oral gavage of an equivalent volume of water for 8 weeks	Anesthetized with sodium pentobarbital (100 mg/kg, intraperitoneally)	1, 2, 3, 4, 7, 8, 9, 10	1. p < 0.05 2. p < 0.01 3. p < 0.01 4. p < 0.01 7. p < 0.01 8. p < 0.01 9. p < 0.01 10. p < 0.01
Li et al. (2024a)	C57BL/6 mice	Male	4 weeks	8/8	NR	HFD feeding for 12 weeks	NR	>99%	Prepared in a laboratory at Huazhong University of Science and Technology	Oral gavage of DHM (25 mg/kg) for 6 weeks	Oral gavage of an equivalent volume of water for 6 weeks	NR	3, 4, 7, 8, 9, 10	3. n.s. 4. p < 0.05 7. n.s. 8. n.s. 9. p < 0.05 10. p < 0.05
Liu (2017)	ApoE^−/−^ mice	Male	8 weeks	10/10	18–22 g	HFD feeding for 12 weeks	Containing 0.3% cholesterol and 20% fat	>98%	Purchased from Shanghai Ronghe Pharmaceutical Technology Development Co., Ltd., (China)	Oral gavage of DHM (100 mg/kg) for 12 weeks	Oral gavage of an equivalent volume of 0.5% CMC-Na solution for 12 weeks	Anesthetized with ether	3, 4, 7, 8, 9, 10	3. n.s. 4. n.s. 7. n.s. 8. n.s. 9. p < 0.01 10. p < 0.01
Liu et al. (2023a)	LDLR^−/−^ mice	Male	4 weeks	8/8	NR	High-fat and high-fructose diet feeding for 12 weeks	Containing 20% fat, 0.5% cholesterol, and 35% fructose	98%	Purchased from Jiangsu Enming Bioengineering Technology Co. Ltd., (China)	Oral gavage of DHM (250 mg/kg) for 12 weeks	Oral gavage of an equivalent volume of 0.5% CMC-Na solution for 6 weeks	Anesthetized with sodium pentobarbital (80 mg/kg, intraperitoneally).	1, 3, 4, 5, 6, 7, 8, 9, 10, 11, 12	1. p < 0.01 3. p < 0.01 4. p < 0.01 5. p < 0.05 6. p < 0.01 7. n.s. 8. p < 0.01 9. p < 0.01 10. p < 0.001 11. n.s. 12. p < 0.05
Ma et al. (2018a)	C57BL/6 mice	Male	6 weeks	15/15	19.7 ± 1.0 g	HFD feeding for 12 weeks	Commercial standard feed (Model H10045) containing 35% carbohydrates, 20% protein, and 45% fat	99%	Purchased from Chengdu Mansite Bio-Technology (China)	Oral gavage of DHM (100 mg/kg) for 12 weeks	Oral gavage of an equivalent volume of PBS for 12 weeks	Anesthetized with ether	2, 3, 4, 7, 8, 11, 12	2. p < 0.05 3. n.s. 4. p < 0.05 7. p < 0.05 8. n.s. 11. p < 0.05 12. n.s.
Ma et al. (2018b)	C57BL/6 mice	Male	6 weeks	50/50	19–21 g	HFD feeding for 12 weeks	Commercial standard feed (Model H10045) containing 46% fat, 36% carbohydrates, and 22% protein.	99%	Purchased from Chengdu Mansite Bio-Technology (China)	Oral gavage of DHM (100 mg/kg) for 12 weeks	Oral gavage of an equivalent volume of PBS for 12 weeks	NR	6	6. p < 0.05
Miao et al. (2023)	C57BL/6 mice	Male	6–8 weeks	7/7	NR	MCD feeding for 8 weeks	No high-fat diet was used	≥98%	Purchased from Shanghai Source Leaf Biological Technology Co., Ltd., (China)	Oral gavage of DHM (300 mg/kg) for 8 weeks	Oral gavage of an equivalent volume of 0.5% CMC-Na solution for 8 weeks	NR	2, 6, 9, 10	2. n.s. 6. p < 0.05 9. p < 0.05 10. n.s.
Song et al. (2022)	C57BL/6 mice	Male	3–4 weeks	10/10	NR	HFD feeding for 4 weeks combined with intraperitoneal injection of streptozotocin for 5 days.	NR	>99%	Purchased from Nanjing Zelang Biotechnology Co., Ltd., (China)	DHM (1,000 mg/kg) mixed into the feed	Control group (no treatment)	NR	2, 3, 4, 5, 6, 7, 8, 9, 10	2. n.s. 3. p < 0.05 4. p < 0.05 5. p < 0.05 6. p < 0.05 7. n.s. 8. p < 0.05 9. p < 0.05 10. p < 0.05
Yang et al. (2024b)	SD rat	Male	8 weeks	7/7	200 ± 20 g	HFD feeding for 8 weeks	Commercial standard feed (Model D12492) containing 60% kcal from fat, 20% kcal from carbohydrates, and 20% kcal from protein	NR	NR	Oral gavage of DHM (200 mg/kg) for 8 weeks	Oral gavage of an equivalent volume of 0.5% CMC-Na solution for 8 weeks	NR	1, 2, 3, 4, 7, 8, 9, 10, 11, 12	1. p < 0.01 2. p < 0.05 3. p < 0.01 4. p < 0.01 7. p < 0.01 8. p < 0.01 9. p < 0.01 10. p < 0.01 11. p < 0.01 12. p < 0.01
Zeng et al. (2019)	SIRT3KO mice	Male	8 weeks	5/5	NR	HFD feeding for 12 weeks	Experimental diet (Model D12451) containing 45% kcal from fat	≥98%	Purchased from Chengdu Mansite Bio-Technology, (China)	HFD supplemented with 0.6% DHM (300 mg/kg)	Control group (no treatment)	Anesthetized with sodium pentobarbital (30 mg/kg, intraperitoneally).	2, 3, 4, 6, 7, 8, 9	2. n.s. 3. n.s. 4. n.s. 6. n.s. 7. n.s. 8. n.s. 9. n.s.
Zeng et al. (2019)	WT mice	Male	8 weeks	5/5	NR	HFD feeding for 12 weeks	Experimental diet (Model D12451) containing 45% kcal from fat	≥98%	Purchased from Chengdu Mansite Bio-Technology, (China)	HFD supplemented with 0.6% DHM (300 mg/kg)	Control group (no treatment)	Anesthetized with sodium pentobarbital (30 mg/kg, intraperitoneally).	2, 3, 4, 6, 7, 8, 9	2. p < 0.001 3. p < 0.05 4. p < 0.05 6. p < 0.01 7. n.s. 8. p < 0.001 9. n.s.
Zeng et al. (2020)	LDLR^−/−^ mice	Male	12 months	8/8	NR	HFD feeding for 12 weeks	Commercial standard feed (Model D12079B) containing 21% fat and 0.21% cholesterol	>98%	Purchased from XI’AN natural field bio-technioue Co., Ltd., (China)	Oral gavage of DHM (400 mg/kg) for 12 weeks	Oral gavage of an equivalent volume of normal saline for 12 weeks	Anesthetized with sodium pentobarbital (50 mg/kg, intraperitoneally).	1, 3, 4, 5, 6, 7, 8, 9, 10, 11, 12	1. p < 0.05 3. p < 0.05 4. p < 0.05 5. p < 0.05 6. p < 0.05 7. p < 0.05 8. p < 0.05 9. p < 0.05 10. p < 0.05 11. p < 0.05 12. p < 0.05
Zhu et al. (2018)	ApoE^−/−^ mice	Male	6 weeks	8/8	NR	HFD feeding for 8 weeks	Commercial standard feed (Model D12079B) containing 21% fat and 0.21% cholesterol	NR	Purchased from Xi’an Tianfeng Biotechnology Co., Ltd., (China)	Oral gavage of DHM (500 mg/kg) for 8 weeks	Oral gavage of an equivalent volume of 0.5% CMC-Na solution for 8 weeks	NR	5, 6	5. p < 0.05 6. p < 0.05

1. body weight, 2. liver index, 3. serum TC, 4. serum TG, 5. TC levels in liver tissue, 6. TG levels in liver tissue, 7. HDL-C, 8. LDL-C, 9. ALT, 10. AST, 11. fasting blood glucose, 12. fasting insulin levels. n.s., not significant; NR, not reported.

**FIGURE 2:**
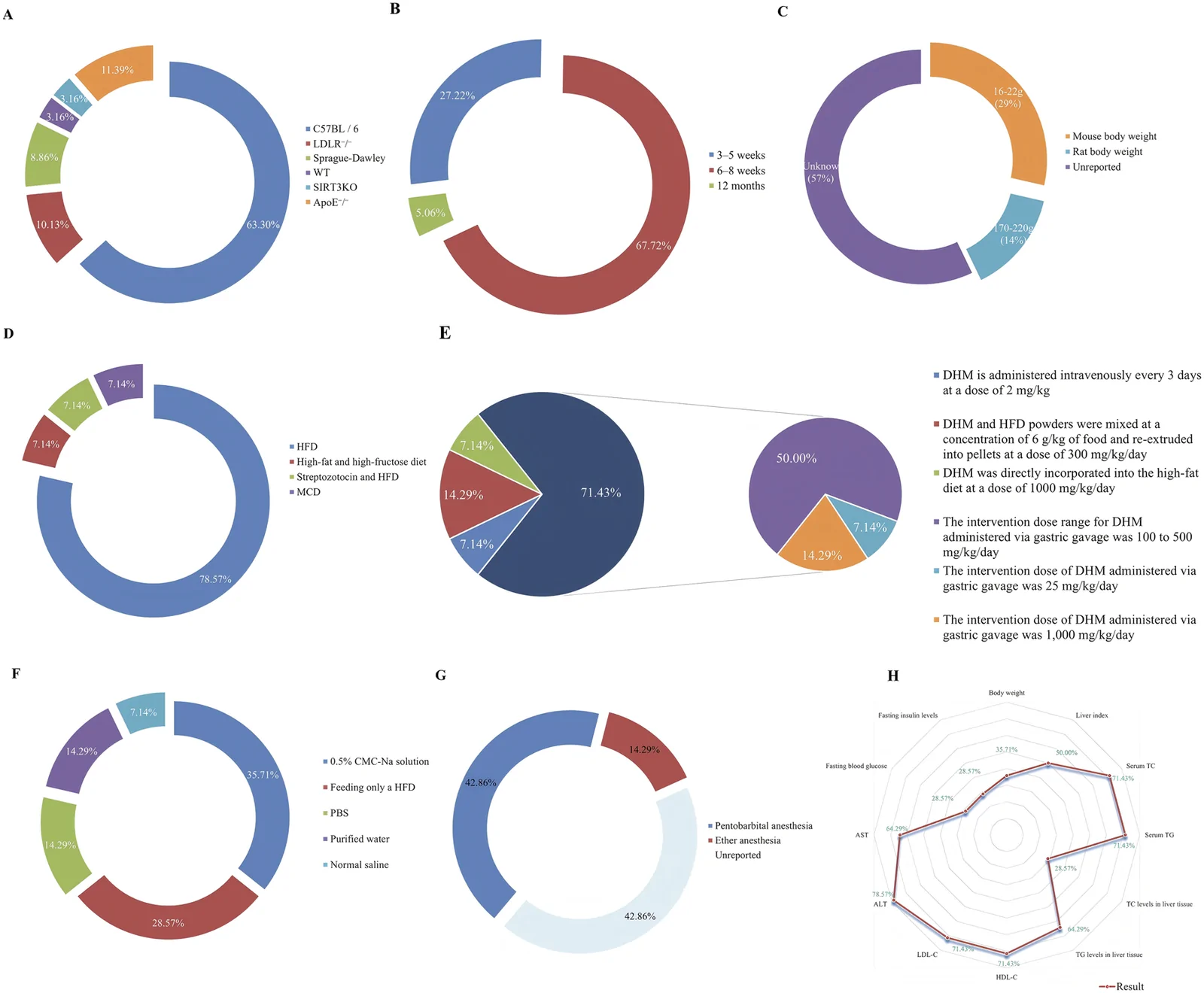
Study characteristics of eligible studies. Distribution of **(A)** experimental models, **(B)** age, **(C)** weight, **(D)** methods of model establishment, **(E)** method of administration, **(F)** control group, **(G)** anesthesia Methods, and **(H)** reporting of Outcome Measures.

In total, 316 animals were used across all studies (158 in experimental groups and 158 in control groups), involving six experimental models (Figure 2A): C57BL/6 mice (n = 200), Low-density lipoprotein receptor knockout (LDLR^−/−^)mice (n = 32), Sprague-Dawley (SD) rats (n = 28), 129 strain wild-type (WT) mice (n = 10), SIRT3 knockout (SIRT3KO) mice (n = 10), and Apolipoprotein E knockout (ApoE^−/−^) mice (n = 36). The sex (male, n = 316) and age (3–5 weeks, n = 86; 6–8 weeks, n = 214; 12 months, n = 16) of the animals varied across experiments (Figure 2B). In most studies, 5–15 animals were used per group; however, one study (Ma et al., 2018b) included 50 mice per group. The body weight of mice ranged from 16 to 22 g, and that of rats ranged from 170 to 220 g. Eight studies (Li et al., 2024a; Liu et al., 2023a; Miao et al., 2023; Song et al., 2022; Zeng et al., 2019; Zeng et al., 2020; Zhu et al., 2018) did not report the animal’s initial body weight (Figure 2C).

A total of eleven studies established NAFLD models by feeding animals a high-fat diet (HFD) for 4–12 weeks. One study (Liu et al., 2023a) induced NAFLD using a high-fat and high-fructose diet administered for 12 weeks. One study (Song et al., 2022) established the model through HFD feeding for 4 weeks combined with intraperitoneal injection of streptozotocin for 5 days. One study (Miao et al., 2023) employed a methionine- and choline-deficient (MCD) diet to induce NAFLD (Figure 2D). According to the predominant pathophysiological characteristics of the experimental models, the studies were classified and interpreted separately as metabolically driven, fibrosis-dominant, diabetic, and genetically modified models. Among them, seven studies were categorized as metabolism-driven models (Guo et al., 2019; Kang et al., 2024; Li et al., 2024a; Ma et al., 2018a; Ma et al., 2018b; Yang et al., 2024c; Zeng et al., 2019), one as a fibrosis-dominant model (Miao et al., 2023), one as a diabetic model (Song et al., 2022), and five as genetically modified models (Liu, 2017; Liu et al., 2023a; Zeng et al., 2019; Zeng et al., 2020; Zhu et al., 2018).

Ten studies (Kang et al., 2024; Li et al., 2024a; Liu, 2017; Liu et al., 2023a; Ma et al., 2018a; Ma et al., 2018b; Miao et al., 2023; Yang et al., 2024c; Zeng et al., 2020; Zhu et al., 2018) administered DHM via gavage, with intervention doses ranging from 25 to 1,000 mg/kg/day. One study (Guo et al., 2019) employed intravenous injection of DHM every 3 days at a dose of 2 mg/kg, two studies (Zeng et al., 2019) mixed DHM with HFD powder at a concentration of 6 g/kg of food, re-pressed into pellets, with a dose of 300 mg/kg/day. One study (Song et al., 2022) incorporated DHM directly into the HFD feed at a dose of 1,000 mg/kg/day (Figure 2E).

Five studies (Liu, 2017; Liu et al., 2023a; Miao et al., 2023; Yang et al., 2024c; Zhu et al., 2018) used an equivalent volume of 0.5% carboxymethyl cellulose sodium (CMC-Na) solution for gavage in the control group, four studies (Guo et al., 2019; Song et al., 2022; Zeng et al., 2019) used a blank control group that was only fed a HFD, two studies (Ma et al., 2018a; Ma et al., 2018b) used an equivalent volume of phosphate-buffered saline (PBS) for gavage, two studies (Kang et al., 2024; Li et al., 2024a) used an equivalent volume of pure water for gavage, and one study (Zeng et al., 2020) used an equivalent volume of normal saline for gavage (Figure 2F).

Six studies (Guo et al., 2019; Kang et al., 2024; Liu et al., 2023a; Zeng et al., 2019; Zeng et al., 2020) used intraperitoneal anesthesia with sodium pentobarbital at doses ranging from 30 to 100 mg/kg, two studies (Ma et al., 2018a; Liu, 2017) used ether anesthesia, and six studies (Li et al., 2024a; Ma et al., 2018b; Miao et al., 2023; Song et al., 2022; Yang et al., 2024c; Zhu et al., 2018) did not report the use of any anesthetic agents (Figure 2G).

A total of twelve outcome indicators were reported across the included studies: five studies reported body weight, seven studies reported liver-to-body weight ratio (%), ten studies reported serum TC, ten studies reported serum TG, four studies reported TC levels in liver tissue, nine studies reported TG levels in liver tissue, ten studies reported HDL-C, ten studies reported LDL-C, eleven studies reported ALT, nine studies reported AST, four studies reported fasting blood glucose, and four studies reported fasting insulin levels (Figure 2H).

### Quality assessment

**FIGURE 3:**
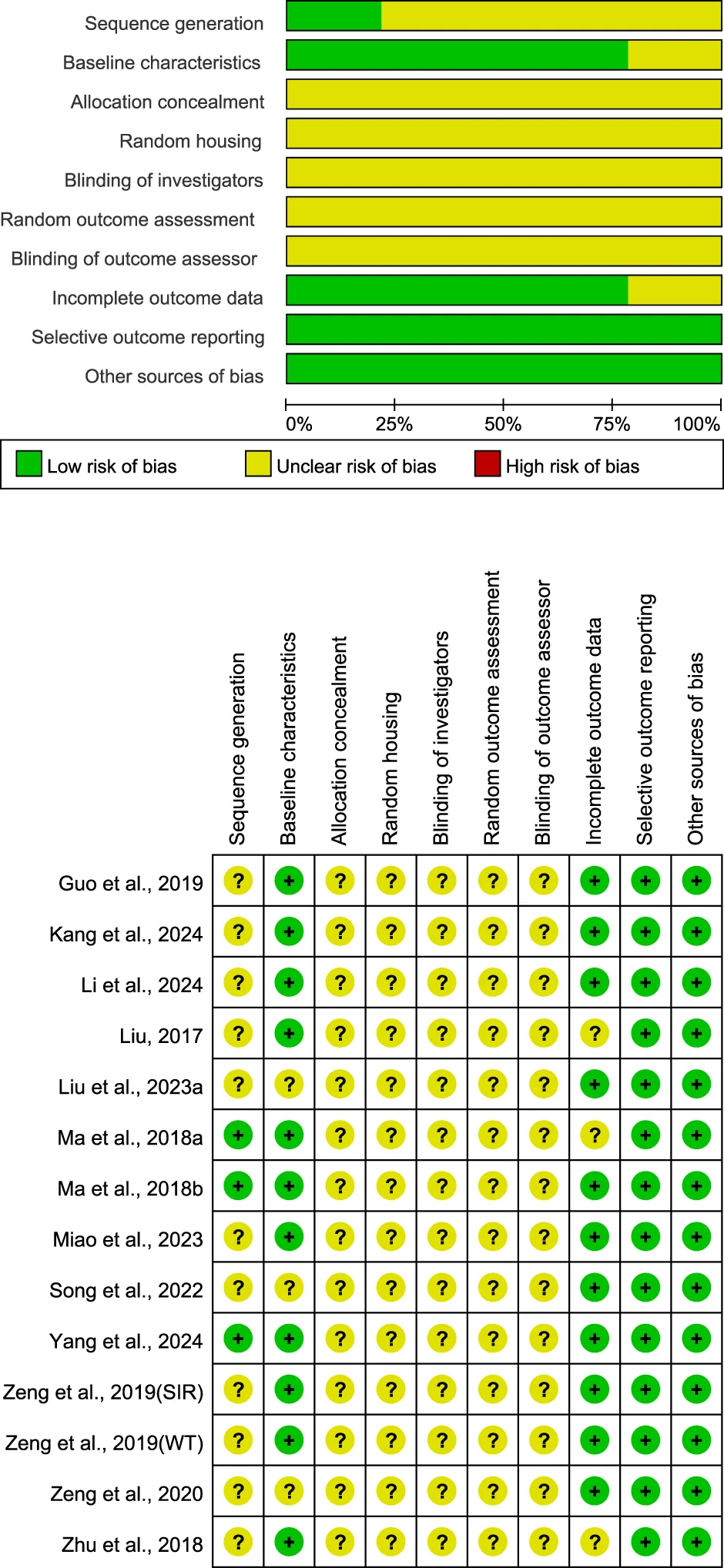
Summary of risk of bias assessment for included studies.

The methodological quality of the included studies is presented in Figure 3. Following the SYRCLE’s RoB tool, the investigators assessed the risk of bias for each animal experiment and classified the studies as having a low, high, or unclear risk of bias. The evaluation domains included the selection of reported results, the measurement of outcomes, missing outcome data, deviations from intended interventions, and the randomization process.

### Primary outcomes

#### Effect of DHM on body weight

**FIGURE 4:**
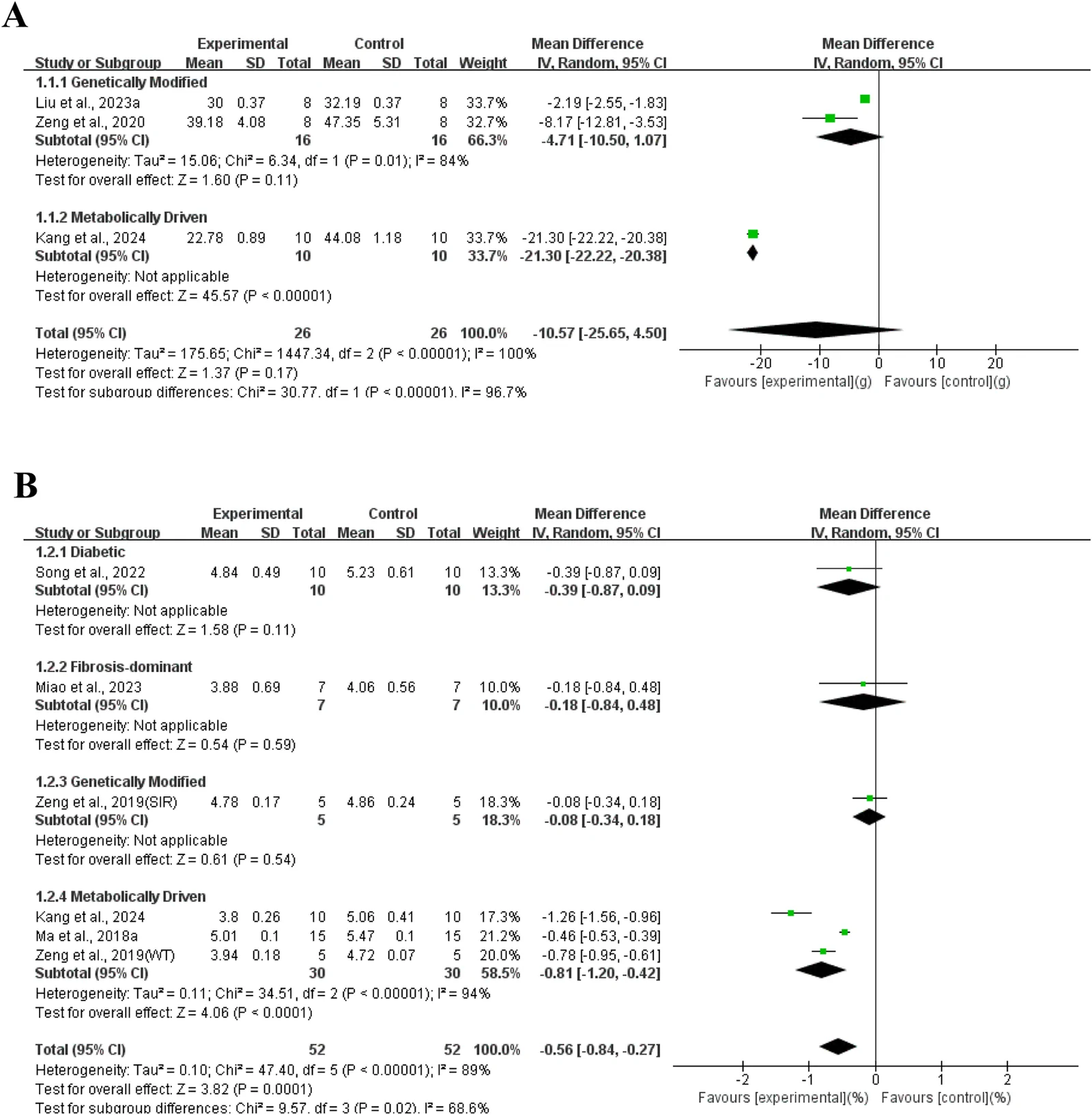
Forest plot showing the effect of DHM treatment on **(A)** body weight (g) and **(B)** liver-to-body weight ratio (%) in animal models.

Five studies (Guo et al., 2019; Kang et al., 2024; Liu et al., 2023a; Yang et al., 2024c; Zeng et al., 2020) were included to evaluate the effects of DHM on body weight in animal models (Figure 4A). Two studies (Guo et al., 2019; Yang et al., 2024c) in rats reported a decrease in body weight following DHM intervention. The mouse studies showed substantial heterogeneity (*p* < 0.00001, I^2^ = 100%); therefore, a random-effects model was applied. Pooled analysis indicated that compared with the model group, a trend toward reduced body weight was observed following DHM treatment. However, the difference did not reach statistical significance (MD = −10.57, 95% CI −25.65 to 4.50, *p* = 0.17).

Subgroup analyses were performed according to the experimental model. In the genetically modified models (Liu et al., 2023a; Zeng et al., 2020), DHM did not significantly reduce body weight (*p* = 0.11), and substantial within-subgroup heterogeneity was observed (*p* = 0.01, I^2^ = 84%). In a metabolically driven model, as represented by a single study (Kang et al., 2024), DHM reduced body weight in mice (MD = −21.30, 95% CI −22.22 to −20.38; *p* < 0.00001).

#### Effect of DHM on liver-to-body weight ratio (%)

Seven studies (Kang et al., 2024; Ma et al., 2018a; Miao et al., 2023; Song et al., 2022; Yang et al., 2024c; Zeng et al., 2019) were included to examine the effects of DHM on the liver-to-body weight ratio (%) in animal models (Figure 4B). One rat study (Yang et al., 2024c) reported a downward trend in liver index following DHM administration. In mouse studies, substantial heterogeneity was observed (*p* < 0.00001, I^2^ = 89%); therefore, a random-effects model was applied. Overall, DHM treatment significantly reduced the liver-to-body weight ratio compared with the model group (MD = −0.56, 95% CI −0.84 to −0.27, *p* = 0.0001).

In the diabetic model (Song et al., 2022), DHM reduced liver index, but the difference was not statistically significant (MD = −0.39, 95% CI −0.87 to 0.09, *p* = 0.11). In the fibrosis-dominant model (Miao et al., 2023), DHM also showed a decreasing trend, but the difference was not significant (MD = −0.18, 95% CI −0.84 to 0.48, *p* = 0.59). In the genetically modified model (Zeng et al., 2019), the effect of DHM was minimal and not significant (MD = −0.08, 95% CI −0.34 to 0.18, *p* = 0.54). Since these three subgroups were each represented by a single study, the evidence is limited and the corresponding results should be interpreted as preliminary observations. In the metabolically driven model (Kang et al., 2024; Ma et al., 2018a; Zeng et al., 2019), DHM significantly reduced the liver-to-body weight ratio (MD = −0.81, 95% CI −1.20 to −0.42, *p* < 0.0001); however, substantial heterogeneity was observed within this subgroup (*p* < 0.00001, I^2^ = 94%).

#### Effect of DHM on serum TC

**FIGURE 5:**
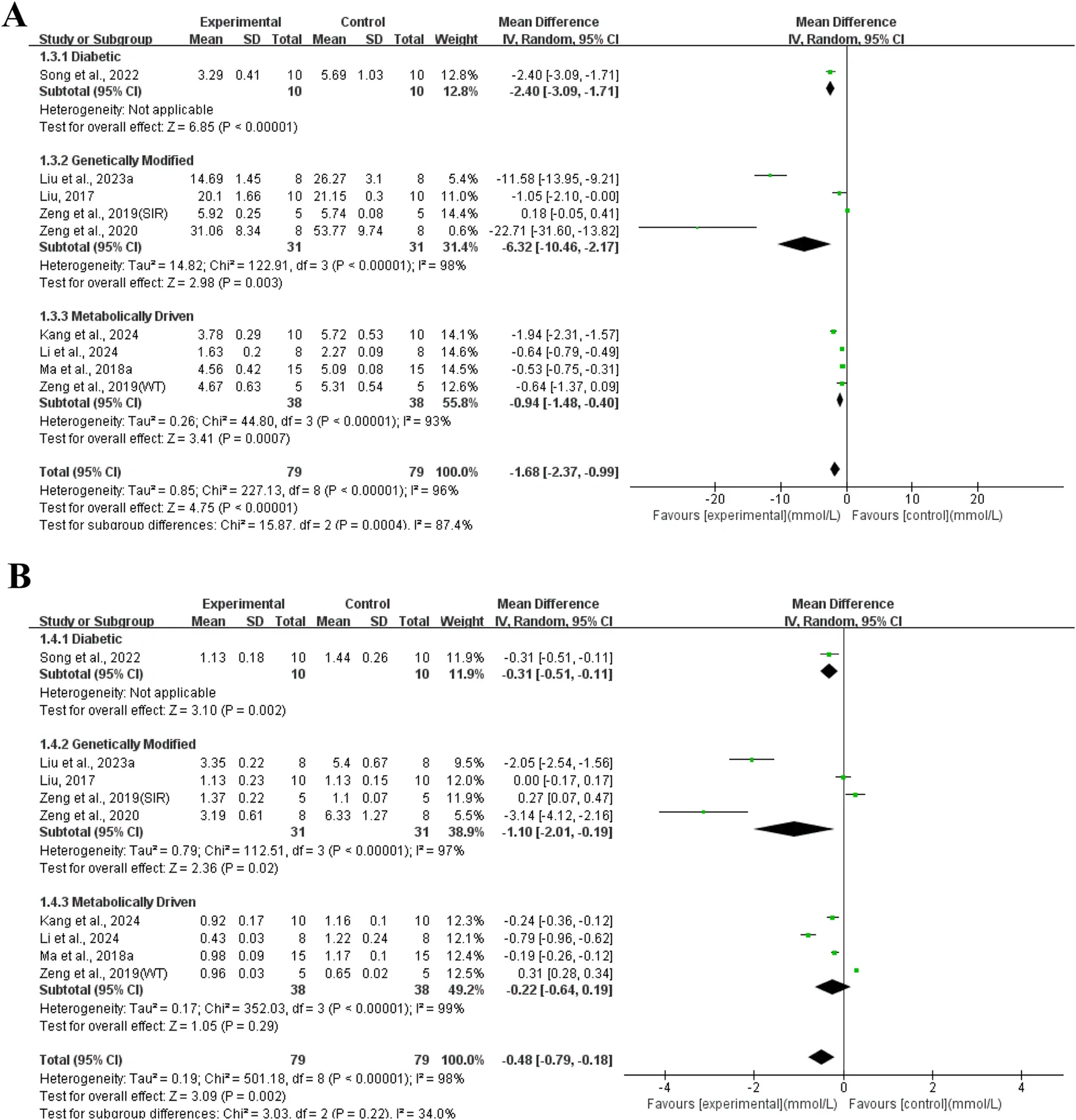
Forest plot showing the effect of DHM treatment on **(A)** serum TC (mmol/L) and **(B)** TG (mmol/L) levels in animal models.

Ten studies (Kang et al., 2024; Li et al., 2024a; Liu, 2017; Liu et al., 2023a; Ma et al., 2018a; Song et al., 2022; Yang et al., 2024c; Zeng et al., 2019; Zeng et al., 2020) were included to evaluate the effects of DHM on serum TC levels in animal models (Figure 5A). One rat study (Yang et al., 2024c) reported that DHM reduced serum TC levels. Given the substantial heterogeneity among mouse studies (*p* < 0.00001, I^2^ = 96%), a random-effects model was employed. The pooled analysis demonstrated that DHM decreased serum TC levels (MD = −1.68, 95% CI −2.37 to −0.99; *p* < 0.00001).

In a diabetic model study (Song et al., 2022), DHM was reported to reduce serum testosterone levels (MD = −2.40, 95% CI −3.09 to −1.71; *p* < 0.00001). In the genetically modified models (Liu, 2017; Liu et al., 2023a; Zeng et al., 2019; Zeng et al., 2020), DHM was also associated with a reduction in TC (MD = −6.32, 95% CI −10.46 to −2.17; *p* = 0.003); however, considerable heterogeneity was present within this subgroup (*p* < 0.00001, I^2^ = 98%). The variation in effect estimates suggests that responsiveness to DHM may differ substantially among genetically modified strains. Similarly, a reduction in serum TC was observed in metabolically driven models (Kang et al., 2024; Li et al., 2024a; Ma et al., 2018a; Zeng et al., 2019) (MD = −0.94, 95% CI −1.48 to −0.40; *p* = 0.0007), although substantial heterogeneity remained (*p* < 0.00001, I^2^ = 93%).

#### Effects of DHM on serum TG

Ten studies (Kang et al., 2024; Li et al., 2024a; Liu, 2017; Liu et al., 2023a; Ma et al., 2018a; Song et al., 2022; Yang et al., 2024c; Zeng et al., 2019; Zeng et al., 2020) were included to investigate the effects of DHM on serum TG levels in animal models (Figure 5B). One rat study (Yang et al., 2024c) reported a downward trend in serum TC levels under DHM treatment. Owing to substantial heterogeneity within mouse studies (*p* < 0.00001, I^2^ = 98%), a random-effects model was applied for pooling. DHM reduced serum TG levels (MD = −0.48, 95% CI −0.79 to −0.18; *p* = 0.002).

Subgroup analyses showed that, in the diabetic model (Song et al., 2022), a reduction in serum TG was observed in the single included study (MD = −0.31, 95% CI −0.51 to −0.11; *p* = 0.002). In the genetically modified model subgroup (Liu, 2017; Liu et al., 2023a; Zeng et al., 2019; Zeng et al., 2020), the pooled effect remained significant (MD = −1.10, 95% CI −2.01 to −0.19; *p* = 0.02), although marked heterogeneity was present within the subgroup (*p* < 0.00001, I^2^ = 97%), suggesting considerable variability in TG responses to DHM across animals with different genetic backgrounds. In the metabolically driven model subgroup (Kang et al., 2024; Li et al., 2024a; Ma et al., 2018a; Zeng et al., 2019), the pooled MD was −0.22 (95% CI −0.64 to 0.19), indicating no significant effect (*p* = 0.29), despite high within-subgroup heterogeneity (*p* < 0.00001, I^2^ = 99%).

#### Effects of DHM on HDL-C

**FIGURE 6:**
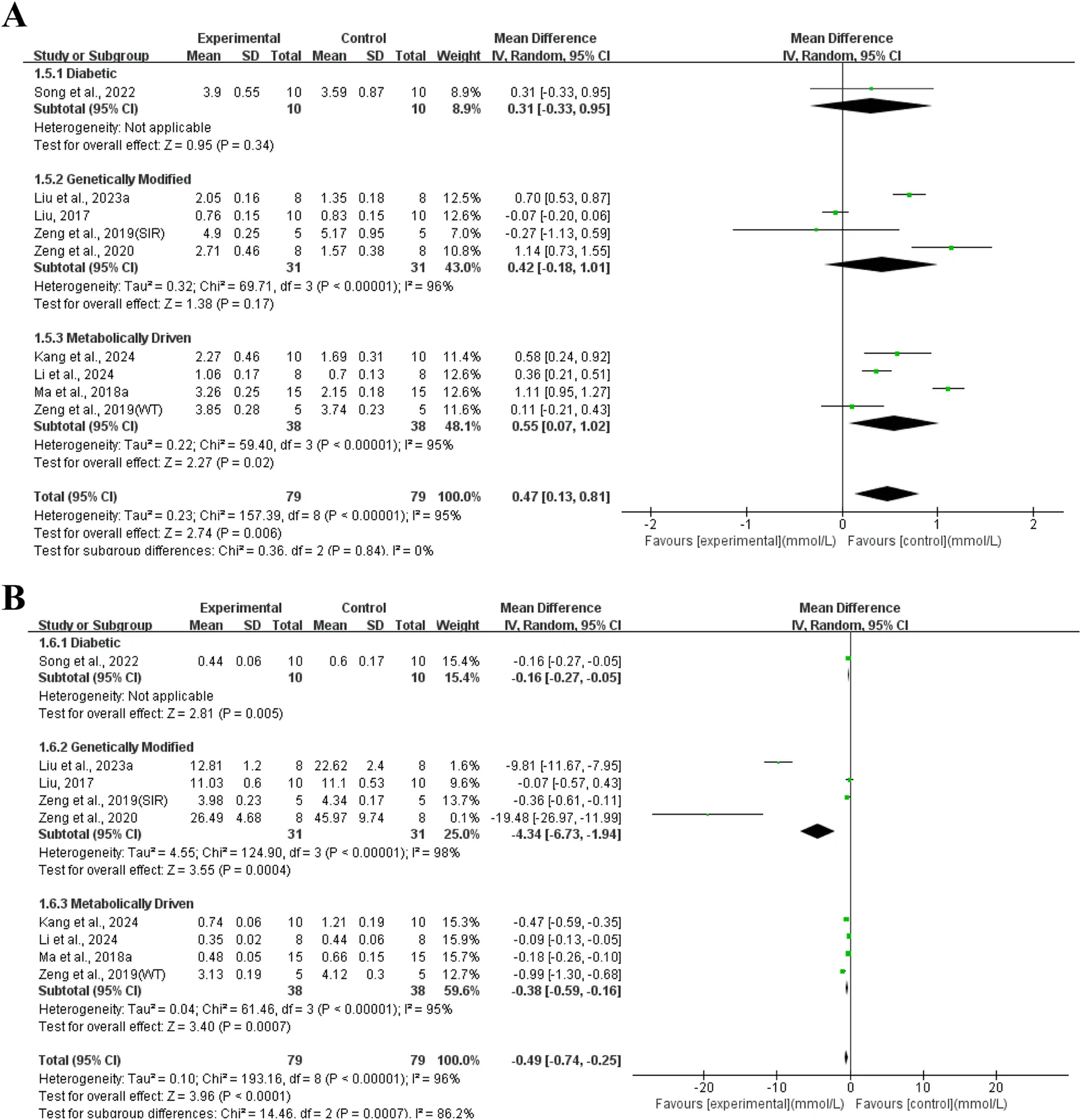
Forest plot showing the effect of DHM treatment on **(A)** HDL-C (mmol/L) and **(B)** LDL-C (mmol/L) levels in animal models.

Ten studies (Kang et al., 2024; Li et al., 2024a; Liu, 2017; Liu et al., 2023a; Ma et al., 2018a; Song et al., 2022; Yang et al., 2024c; Zeng et al., 2019; Zeng et al., 2020) were included to evaluate the effects of DHM on HDL-C levels in animal models (Figure 6A). One rat study (Yang et al., 2024c) reported an increase in serum HDL-C levels following DHM treatment. Because substantial heterogeneity was observed among mouse studies (*p* < 0.00001, I^2^ = 95%), a random-effects model was applied. The pooled analysis showed that DHM improved serum HDL-C levels (MD = 0.47, 95% CI 0.13 to 0.81; *p* = 0.006).

Only one study used a diabetic model (Song et al., 2022) and reported no significant effect on HDL-C (MD = 0.31, 95% CI −0.33 to 0.95; *p* = 0.34). In genetically modified models (Liu, 2017; Liu et al., 2023a; Zeng et al., 2019; Zeng et al., 2020), the pooled estimate did not reach statistical significance (MD = 0.42, 95% CI −0.18 to 1.01; *p* = 0.17), and substantial heterogeneity remained within the subgroup (*p* < 0.00001, I^2^ = 96%). In metabolically driven models (Kang et al., 2024; Li et al., 2024a; Ma et al., 2018a; Zeng et al., 2019), DHM increased HDL-C levels (MD = 0.55, 95% CI 0.07 to 1.02; *p* = 0.02), although considerable heterogeneity persisted (*p* < 0.00001, I^2^ = 95%).

#### Effects of DHM on LDL-C

Ten studies (Kang et al., 2024; Li et al., 2024a; Liu, 2017; Liu et al., 2023a; Ma et al., 2018a; Song et al., 2022; Yang et al., 2024c; Zeng et al., 2019; Zeng et al., 2020) were included to assess the effects of DHM on LDL-C levels in animal models (Figure 6B). One rat study (Yang et al., 2024c) indicated a decline in LDL-C levels with DHM supplementation. Given the marked heterogeneity throughout the included mouse studies (*p* < 0.00001, I^2^ = 96%), a random-effects model was adopted. The pooled estimate indicated that DHM reduced LDL-C levels (MD = −0.49, 95% CI −0.74 to −0.25; *p* < 0.0001).

The diabetic model (Song et al., 2022) stratum included only one study, which reported that DHM was associated with a reduction in LDL-C levels (MD = −0.16, 95% CI −0.27 to −0.05; *p* = 0.005). In genetically modified models (Liu, 2017; Liu et al., 2023a; Zeng et al., 2019; Zeng et al., 2020) the pooled effect favored DHM treatment (MD = −4.34, 95% CI −6.73 to −1.94; *p* = 0.0004). Nevertheless, heterogeneity within this subgroup remained substantial (*p* < 0.00001, I^2^ = 98%). In metabolically driven models (Kang et al., 2024; Li et al., 2024a; Ma et al., 2018a; Zeng et al., 2019), DHM likewise reduced LDL-C concentrations (MD = −0.38, 95% CI −0.59 to −0.16; *p* = 0.0007), although considerable between-study variability persisted (*p* < 0.00001, I^2^ = 95%).

#### Effects of DHM on TC and TG levels in hepatic tissue

**FIGURE 7:**
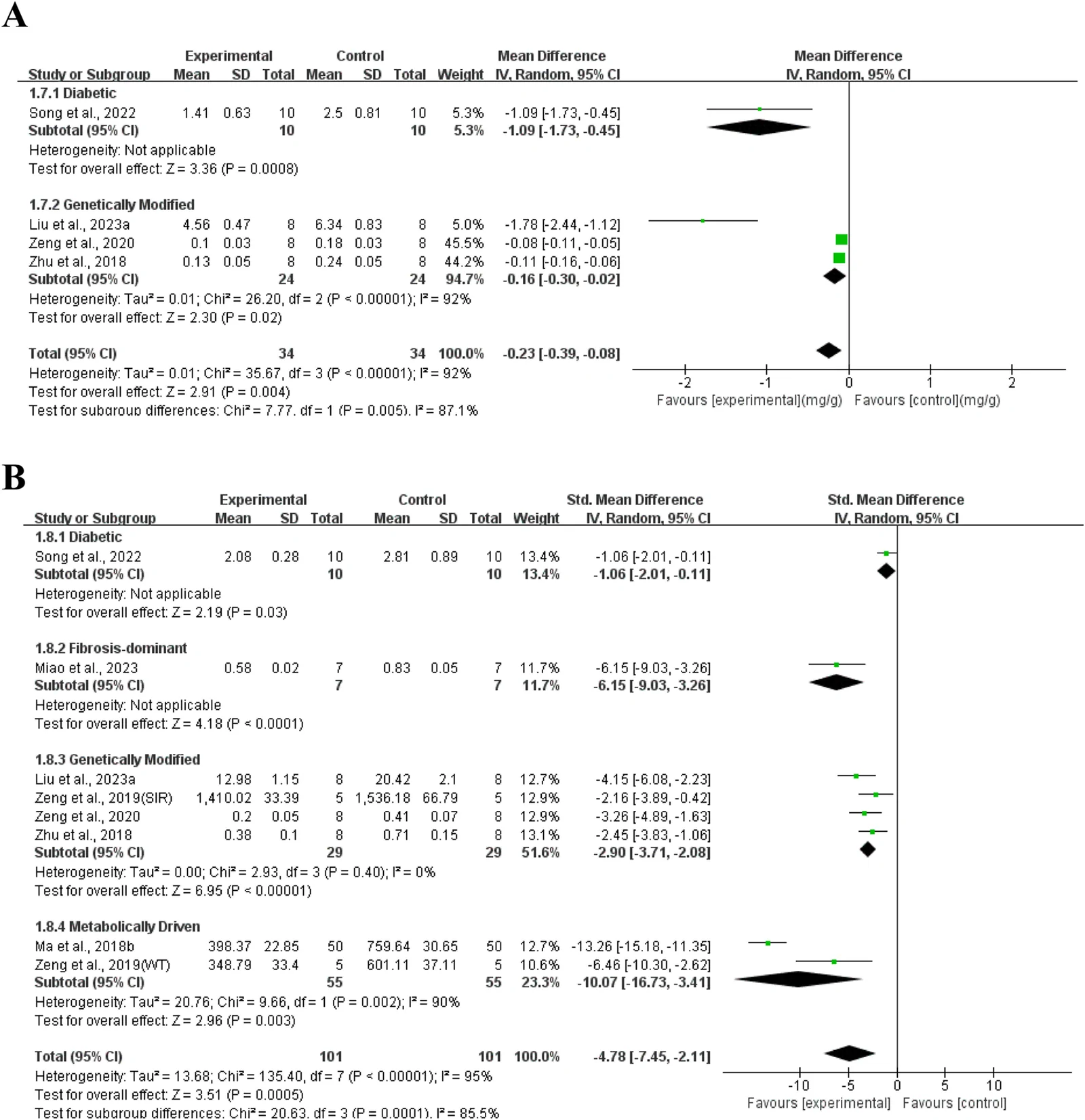
Forest plot showing the effect of DHM treatment on **(A)** TC (mg/g) and **(B)** TG levels in hepatic tissue of animal models.

Four studies (Liu et al., 2023a; Song et al., 2022; Zeng et al., 2020; Zhu et al., 2018) were included to evaluate the effects of DHM on TC levels in liver tissue of animal models (Figure 7A). Given the substantial heterogeneity among studies (*p* < 0.00001, I^2^ = 92%), a random-effects model was used to pool effect estimates. The analysis showed that DHM treatment reduced hepatic TC levels (MD = −0.23, 95% CI −0.39 to −0.08, *p* = 0.004). A single study was conducted in a diabetic model (Song et al., 2022), which showed that DHM reduced hepatic TC (MD = −1.09, 95% CI −1.73 to −0.45, *p* = 0.0008). In the genetically modified model subgroup (Liu et al., 2023a; Zeng et al., 2020; Zhu et al., 2018), the pooled effect favored DHM (MD = −0.16, 95% CI −0.30 to −0.02, *p* = 0.02), although substantial heterogeneity was observed within this subgroup (*p* < 0.00001, I^2^ = 92%).

Nine studies (Guo et al., 2019; Liu et al., 2023a; Ma et al., 2018b; Miao et al., 2023; Song et al., 2022; Zeng et al., 2019; Zeng et al., 2020; Zhu et al., 2018) were included to investigate the effects of DHM on TG levels in liver tissue of animal models (Figure 7B). One rat study (Guo et al., 2019) documented a decrease in hepatic TG content after DHM intervention. As marked inconsistency was detected across mouse studies (*p* < 0.00001, I^2^ = 95%), SMDs were synthesized using a random-effects approach. The combined estimate suggested that DHM attenuated TG levels in hepatic tissue accumulation (SMD = −4.78, 95% CI −7.45 to −2.11; *p* = 0.0005). With respect to the diabetic model (Song et al., 2022), the available evidence comes from only one study which observed a reduction in hepatic TG content with DHM (SMD = −1.06, 95% CI −2.01 to −0.11; *p* = 0.03). For the fibrosis-dominant model (Miao et al., 2023), similarly, only a single study is available, whose results showed a significant decrease in hepatic TG levels after DHM intervention (SMD = −6.15, 95% CI −9.03 to −3.26; *p* < 0.0001). Among genetically modified models (Liu et al., 2023a; Zeng et al., 2019; Zeng et al., 2020; Zhu et al., 2018), the summary effect continued to favor DHM (SMD = −2.90, 95% CI −3.71 to −2.08; *p* < 0.00001), with negligible heterogeneity detected within the subgroup (*p* = 0.40, I^2^ = 0%). In metabolically driven models (Ma et al., 2018b; Zeng et al., 2019), the aggregated SMD was −10.07 (95% CI −16.73 to −3.41; *p* = 0.003); however, variability across studies remained substantial (*p* = 0.002, I^2^ = 90%).

#### Effects of DHM on ALT

**FIGURE 8:**
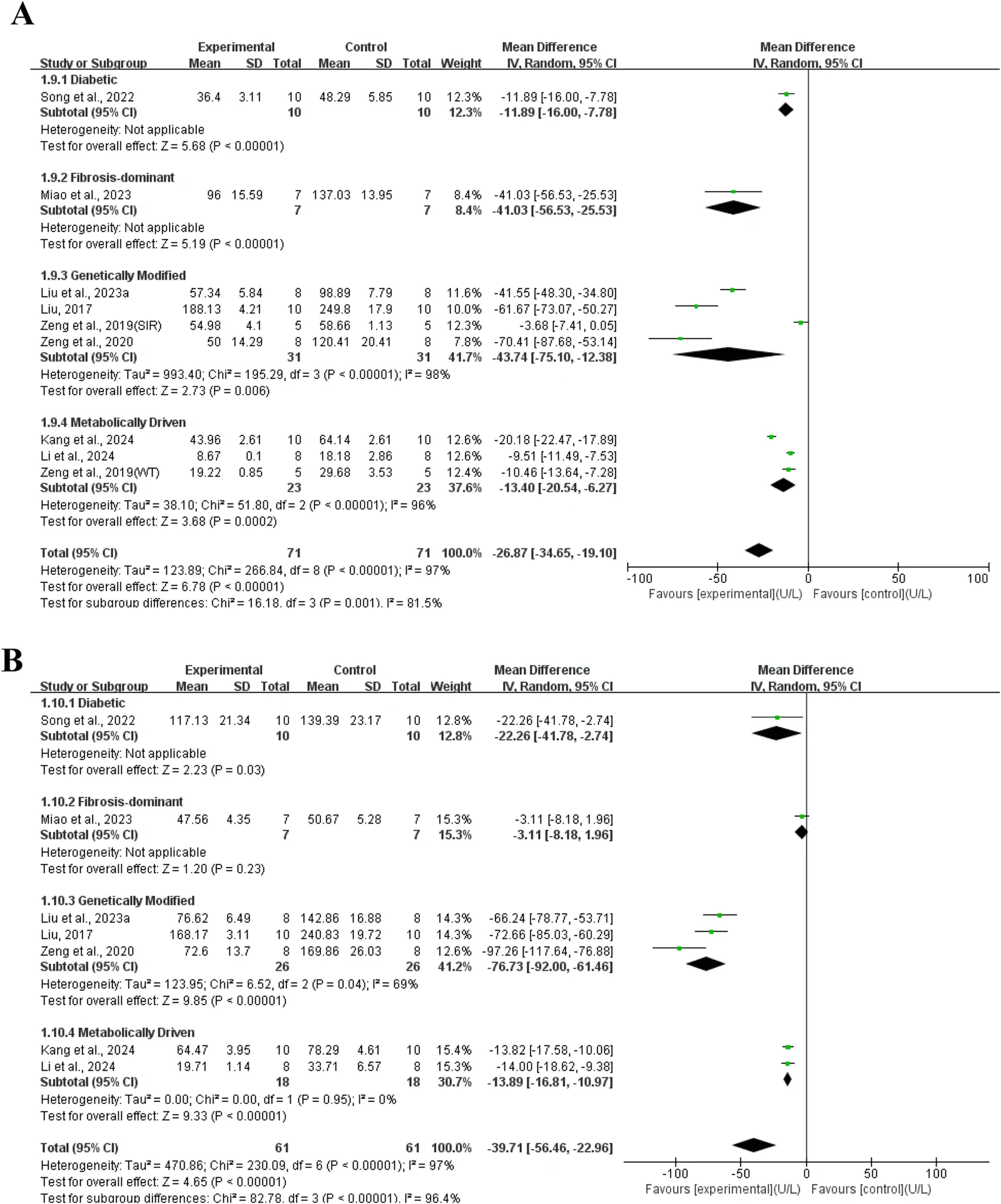
Forest plot showing the effect of DHM treatment on **(A)** ALT (U/L) and **(B)** AST (U/L) levels in animal models.

Eleven studies (Guo et al., 2019; Kang et al., 2024; Li et al., 2024a; Liu, 2017; Liu et al., 2023a; Miao et al., 2023; Song et al., 2022; Yang et al., 2024c; Zeng et al., 2019; Zeng et al., 2020) were included to evaluate the effects of DHM on ALT levels in animal models (Figure 8A). In two rat studies (Guo et al., 2019; Yang et al., 2024c), ALT levels declined in response to DHM treatment. Given the substantial heterogeneity observed across mouse investigations (*p* < 0.00001, I^2^ = 97%), a random-effects model was employed. The pooled estimate indicated that DHM reduced serum ALT levels (MD = −26.87, 95% CI −34.65 to −19.10, *p* < 0.00001).

In the sole diabetic model study (Song et al., 2022) identified, DHM treatment resulted in a decline in ALT (MD = −11.89, 95% CI −16.00 to −7.78; *p* < 0.00001). The one study using a fibrosis-dominant model (Miao et al., 2023), yielded a similarly large reduction (MD = −41.03, 95% CI −56.53 to −25.53; *p* < 0.00001). In genetically modified models (Liu, 2017; Liu et al., 2023a; Zeng et al., 2019; Zeng et al., 2020), the summary estimate also favored DHM (MD = −43.74, 95% CI −75.10 to −12.38; *p* = 0.006), although considerable heterogeneity was evident within this subgroup (*p* < 0.00001, I^2^ = 98%). A comparable reduction in ALT levels was observed in metabolically driven models (Kang et al., 2024; Li et al., 2024a; Zeng et al., 2019) (MD = −13.40, 95% CI −20.54 to −6.27; *p* = 0.0002), despite substantial residual heterogeneity (*p* < 0.00001, I^2^ = 96%).

#### Effects of DHM on AST

Nine studies (Guo et al., 2019; Kang et al., 2024; Li et al., 2024a; Liu, 2017; Liu et al., 2023a; Miao et al., 2023; Song et al., 2022; Yang et al., 2024c; Zeng et al., 2020) were included to assess the effects of DHM on AST levels in animal models (Figure 8B). Two rat studies (Guo et al., 2019; Yang et al., 2024c) consistently reported reductions in AST levels with DHM supplementation. Owing to substantial heterogeneity across the included experiments (*p* < 0.00001, I^2^ = 97%), a random-effects model was used to pool MD. The combined results demonstrated that DHM decreased AST levels (MD = −39.71, 95% CI −56.46 to −22.96; *p* < 0.00001).

The diabetic model (Song et al., 2022) was derived from a single study and showed a reduction in AST levels following DHM treatment (MD = −22.26, 95% CI −41.78 to −2.74; *p* = 0.03). The fibrosis-dominant model (Miao et al., 2023), also derived from a single study, did not yield a statistically significant effect (MD = −3.11, 95% CI −8.18 to 1.96; *p* = 0.23). In genetically modified models (Liu, 2017; Liu et al., 2023a; Zeng et al., 2020), the pooled estimate indicated a pronounced reduction in AST levels (MD = −76.73, 95% CI −92.00 to −61.46; *p* < 0.00001), with moderate heterogeneity observed within the subgroup (*p* = 0.04, I^2^ = 69%). A corresponding effect was observed in metabolically driven models (Kang et al., 2024; Li et al., 2024a), where DHM was associated with lower AST levels (MD = −13.89, 95% CI −16.81 to −10.97; *p* < 0.00001), and no evidence of heterogeneity was detected (*p* = 0.95, I^2^ = 0%).

#### Effects of DHM on fasting blood glucose levels and insulin levels

**FIGURE 9:**
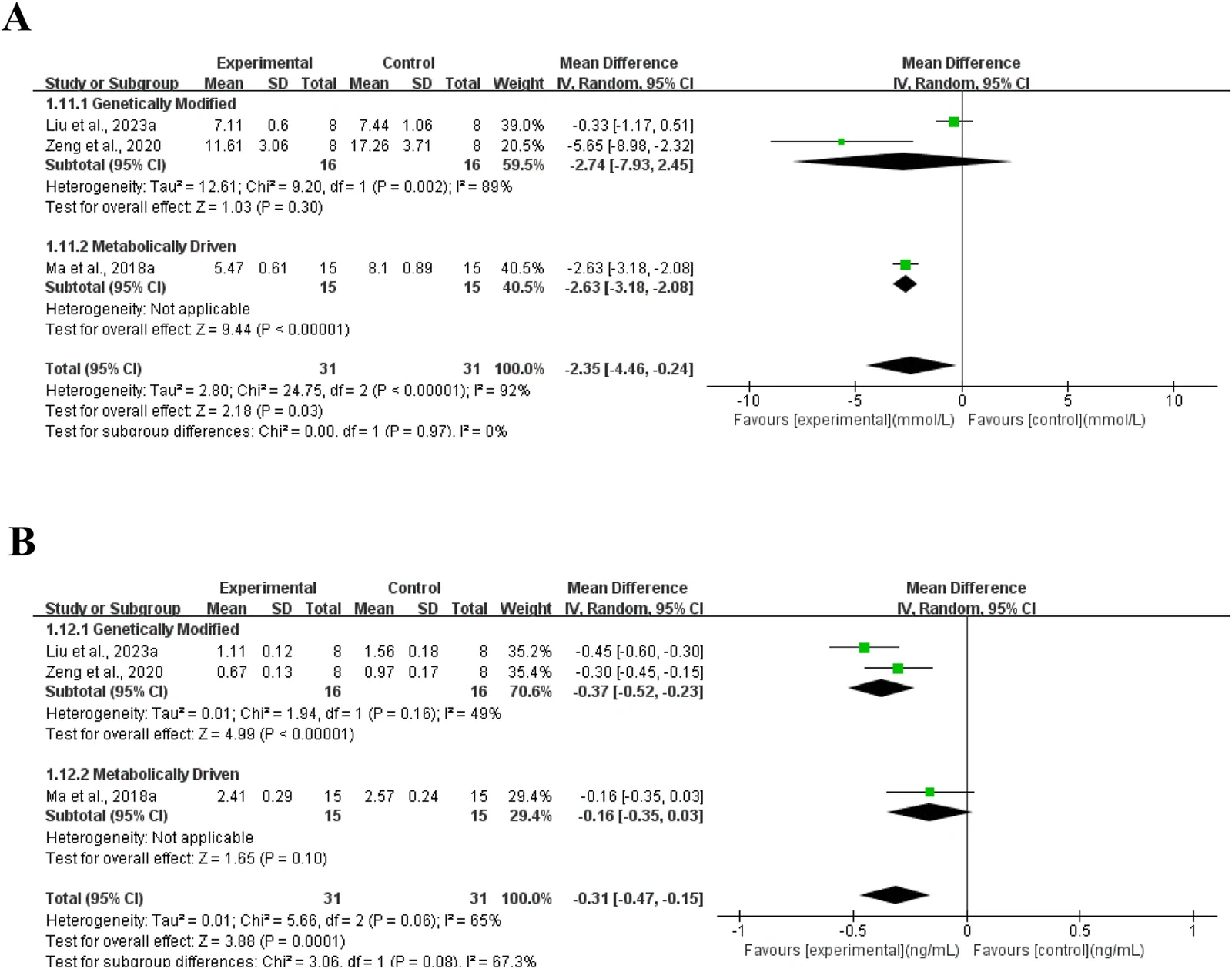
Forest plot showing the effect of DHM treatment on **(A)** fasting blood glucose (mmol/L) and **(B)** insulin (ng/mL) levels in animal models.

Four studies (Liu et al., 2023a; Ma et al., 2018a; Yang et al., 2024c; Zeng et al., 2020) were included to evaluate the effects of DHM on fasting blood glucose levels in animal models (Figure 9A). One rat study (Yang et al., 2024c) reported a reduction in fasting blood glucose levels following DHM intervention. Because substantial heterogeneity was present across the included mouse studies (*p* < 0.00001, I^2^ = 92%), a random-effects model was selected. The overall estimate indicated that DHM was associated with lower fasting blood glucose levels (MD = −2.35, 95% CI −4.46 to −0.24; *p* = 0.03). In genetically modified models (Liu et al., 2023a; Zeng et al., 2020), the pooled effect did not reach statistical significance (MD = −2.74, 95% CI −7.93 to 2.45; *p* = 0.30), and considerable heterogeneity remained within the subgroup (*p* = 0.002, I^2^ = 89%). The metabolically driven (Ma et al., 2018a) subgroup was derived from a single study and demonstrated a reduction in fasting blood glucose levels following DHM treatment (MD = −2.63, 95% CI −3.18 to −2.08; *p* < 0.00001).

Four studies (Liu et al., 2023a; Ma et al., 2018a; Yang et al., 2024c; Zeng et al., 2020) were included to evaluate the effects of DHM on fasting insulin levels in animal models (Figure 9B). In one rat study (Yang et al., 2024c), fasting insulin levels declined following DHM exposure. As moderate heterogeneity was observed across mouse studies (*p* = 0.06, I^2^ = 65%), a random-effects model was implemented for data synthesis. The summary estimate demonstrated that DHM reduced fasting insulin levels (MD = −0.31, 95% CI −0.47 to −0.15; *p* = 0.0001). In genetically modified models (Liu et al., 2023a; Zeng et al., 2020), the pooled MD was −0.37 (95% CI −0.52 to −0.23; *p* < 0.00001), with low between-study heterogeneity (*p* = 0.16, I^2^ = 49%). By contrast, evidence from the metabolically driven (Ma et al., 2018a) subgroup was available from only one study and suggested a reduction in fasting insulin levels that did not reach statistical significance (MD = −0.16, 95% CI −0.35 to 0.03; *p* = 0.10).

### Molecular mechanisms and signaling pathways

The mechanisms underlying DHM’s effects in NAFLD, along with its major molecular targets, are summarized in Table 2. Among the 14 independent experiments included, eleven studies (Guo et al., 2019; Kang et al., 2024; Liu, 2017; Liu et al., 2023a; Ma et al., 2018a; Ma et al., 2018b; Yang et al., 2024c; Zeng et al., 2019; Zeng et al., 2020; Zhu et al., 2018) described detailed molecular mechanisms.

**TABLE 2:** Summary of the major molecular mechanisms and signaling pathways involved in the protective effects of DHM against NAFLD.

Study, year	Molecular pathway	Proteins	Functional clustering
Guo et al. (2019)	NF-κB/p53/Bax	↓NF-κB; ↓p53; ↓Bax	Inhibit apoptosis and inflammation
Kang et al. (2024)	TLR4/NF-κB	↓TLR4; ↓NF-κB p65	Inhibit inflammatory response
Liu (2017)	AMPK	↑p-AMPK	Improve lipid metabolism
Liu et al. (2023a)	PI3K/Akt	↑p-Akt	Improve insulin sensitivity, inhibit liver lipid deposition
Ma et al. (2018a)	Mitochondrial Fusion/Fission	↓Fis1; ↓Drp1; ↑Mfn2; ↑Opa1	Improve mitochondrial function and reduce hepatic steatosis
Ma et al. (2018b)	Circadian Clock Pathway	↑CLOCK, ↑BMAL1, ↑PER2	Restores circadian rhythm of clock genes, improves hepatic lipid metabolism.
Yang et al. (2024b)	AMPK/PGC-1α/PPARα	↑p-AMPK; ↑PGC-1α,↑PPARα	Induce autophagy; Improve lipid metabolism; Ameliorate insulin resistance
Zeng et al. (2019)	AMPK-PGC1α/ERRα-SIRT3	↑SIRT3; ↑p-AMPK; ↑PGC1α	Improve mitochondrial function and redox homeostasis
Zeng et al. (2019)	AMPK-PGC1α/ERRα-SIRT3	↑SIRT3; ↑p-AMPK; ↑PGC1α	Improve mitochondrial function and redox homeostasis
Zeng et al. (2020)	SIRT1/AMPK/NF-κB	↑SIRT1; ↑p-AMPK; ↓Ac-NF-κB	Ameliorate steatosis, inflammation and fibrosis
Zhu et al. (2018)	Cholesterol Reverse Transport	↑ABCA1; ↑ABCG1; ↑ABCG5; ↑ABCG8; ↑CYP7A1	Promote cholesterol efflux and excretion

↑: Up-regulation of protein expression, ↓: Down-regulation of protein expression.

Based on the available evidence, the pharmacological effects of DHM can be summarized as follows: regulation of lipid metabolism (n = 3), reduction of hepatic steatosis (n = 3), inhibition of inflammatory responses (n = 3), improvement of mitochondrial function (n = 3), and amelioration of insulin resistance (n = 2). Among these, modulation of lipid metabolism and reduction of hepatic steatosis were the most frequently reported mechanisms, with these effects described in six studies (Liu, 2017; Liu et al., 2023a; Ma et al., 2018a; Ma et al., 2018b; Yang et al., 2024c; Zeng et al., 2020).

Inhibition of inflammation and apoptosis represented another major mechanism. DHM effectively suppressed the release of inflammatory cytokines and downregulated apoptosis-related signaling pathways, as reported in three studies (Guo et al., 2019; Kang et al., 2024; Zeng et al., 2020). Improvements in mitochondrial function and redox homeostasis were also repeatedly observed, with DHM enhancing mitochondrial respiration and promoting reactive oxygen species (ROS) clearance, as documented in three studies (Ma et al., 2018a; Zeng et al., 2019). Furthermore, amelioration of insulin resistance and activation of autophagy were identified as key processes contributing to DHM’s metabolic regulatory effects, supported by two studies (Liu et al., 2023a; Yang et al., 2024c). In addition, DHM was reported to promote cholesterol efflux and excretion (Zhu et al., 2018), and to restore the rhythmic expression of circadian clock genes (Ma et al., 2018b).

### Influence of dose and treatment duration on the pharmacological effects of DHM

The dose-response and time-effect relationships play a substantial role in clinical medication (Li et al., 2022). To evaluate the influence of dose and treatment duration on the pharmacological effects of DHM, subgroup analyses were conducted for six key metabolic and hepatic outcomes. The stratification thresholds were 100 mg/kg/day for dose and 8 weeks for treatment duration. Integrated 3D plots and dose-response radar charts were subsequently generated as descriptive visualizations of the combined dose-duration patterns. These visualizations were used to explore candidate dose-time trends that may require further evaluation in future well-controlled studies.

#### Serum TC

**FIGURE 10:**
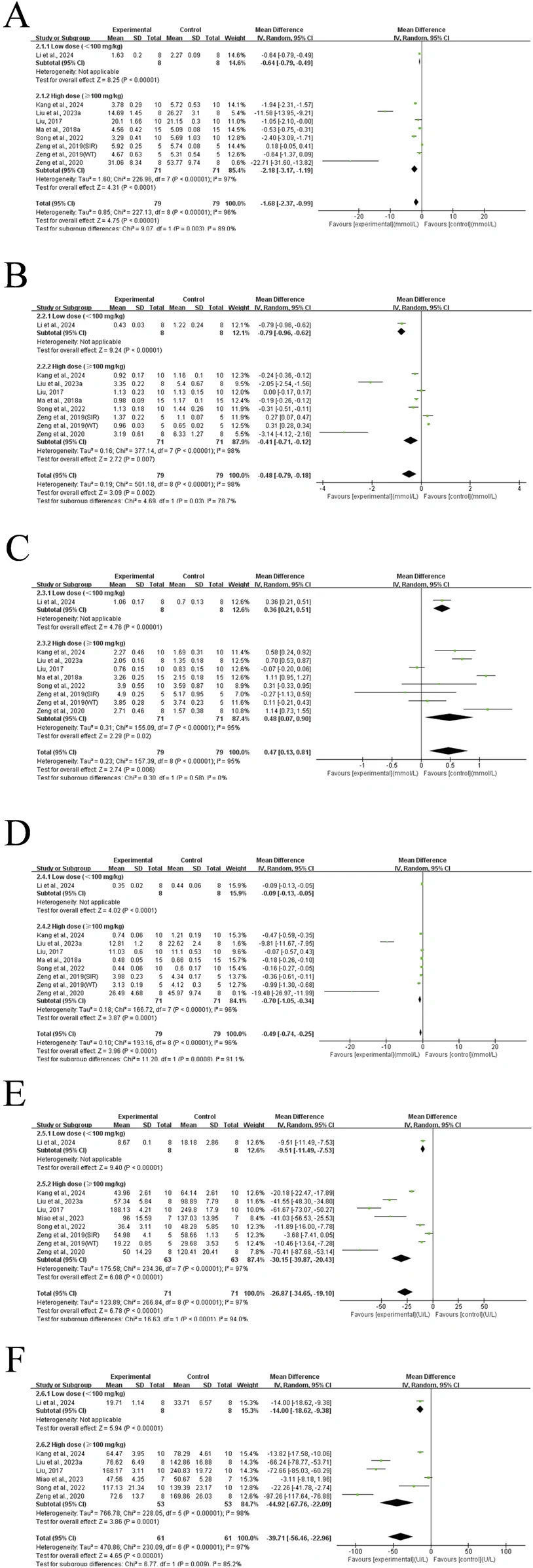
Dose-stratified subgroup analyses of the effects of DHM on **(A)** serum TC, **(B)** TG, **(C)** HDL-C, **(D)** LDL-C, **(E)** ALT, and **(F)** AST.

**FIGURE 11:**
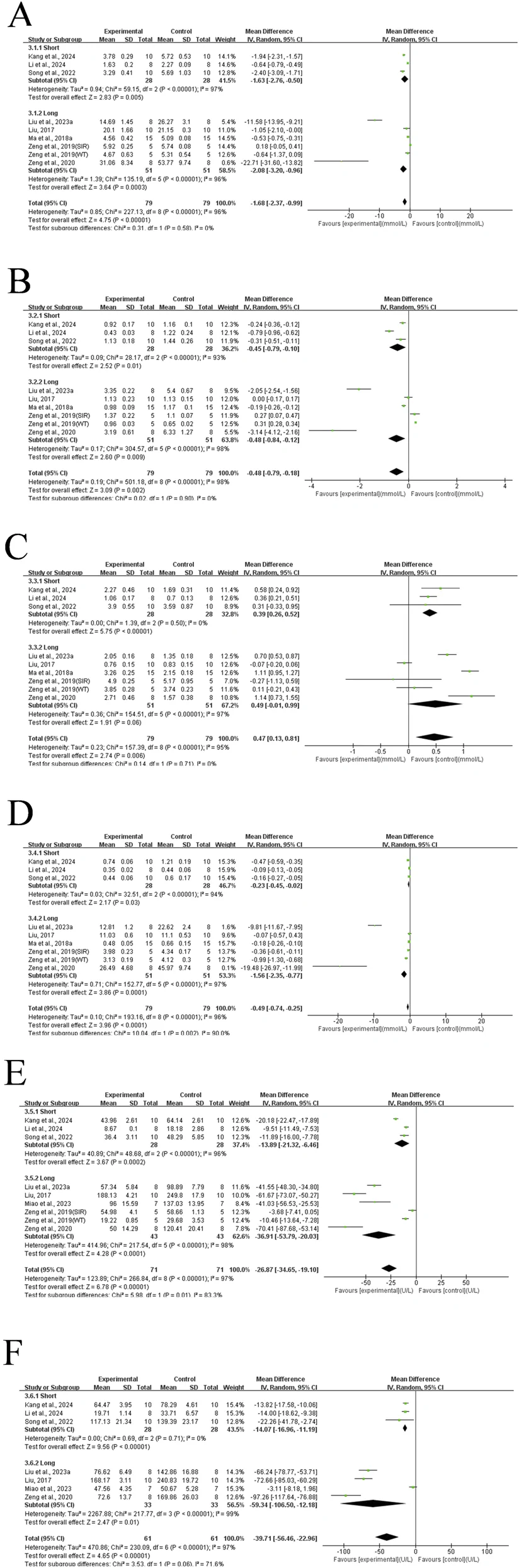
Duration-stratified subgroup analyses of the effects of DHM on **(A)** serum TC, **(B)** TG, **(C)** HDL-C, **(D)** LDL-C, **(E)** ALT, and **(F)** AST.

**FIGURE 12:**
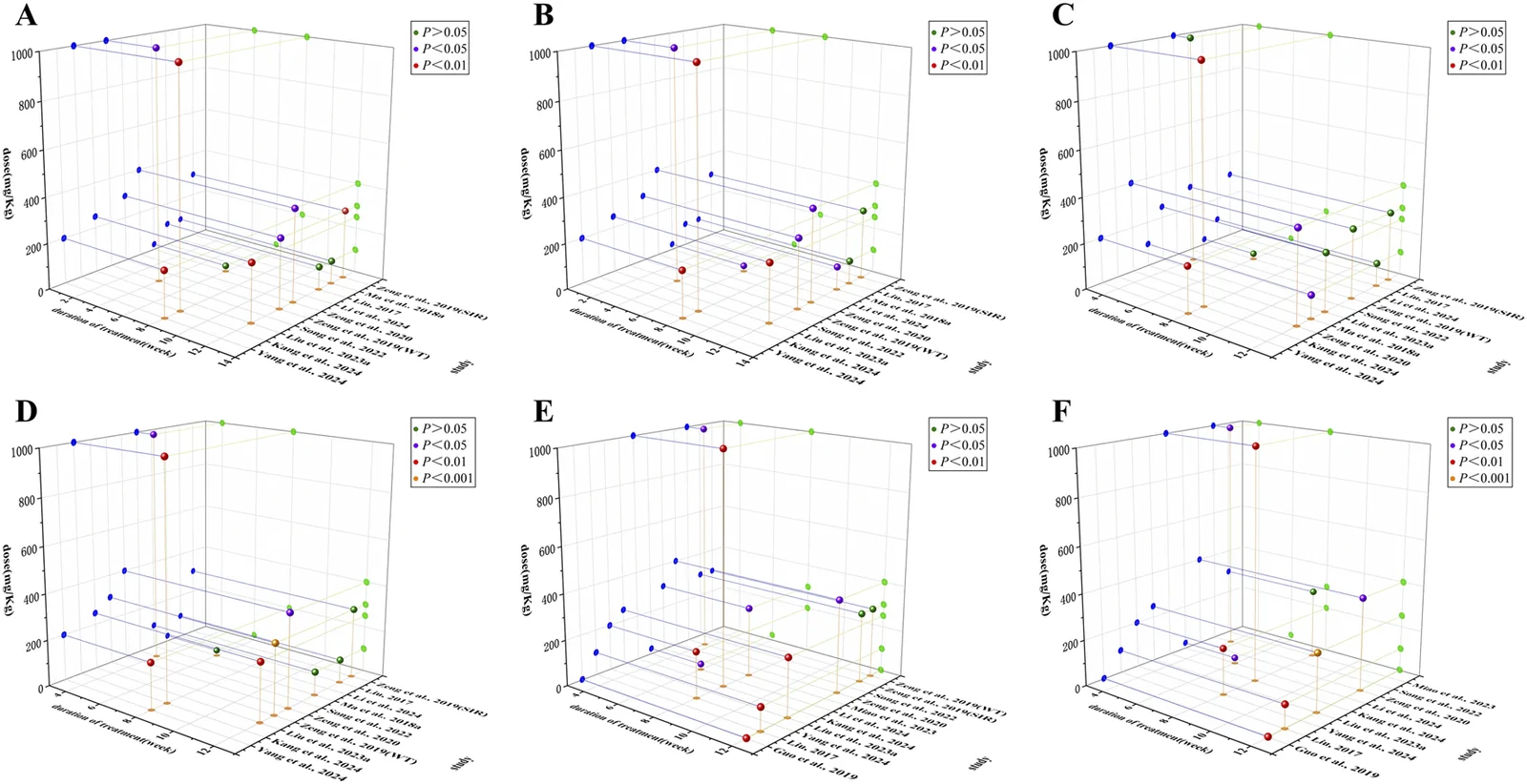
3D dose/time-effect plots of the effects of DHM treatment on **(A)** serum TC, **(B)** serum TG, **(C)** HDL-C, **(D)** LDL-C, **(E)** ALT, and **(F)** AST.

**FIGURE 13:**
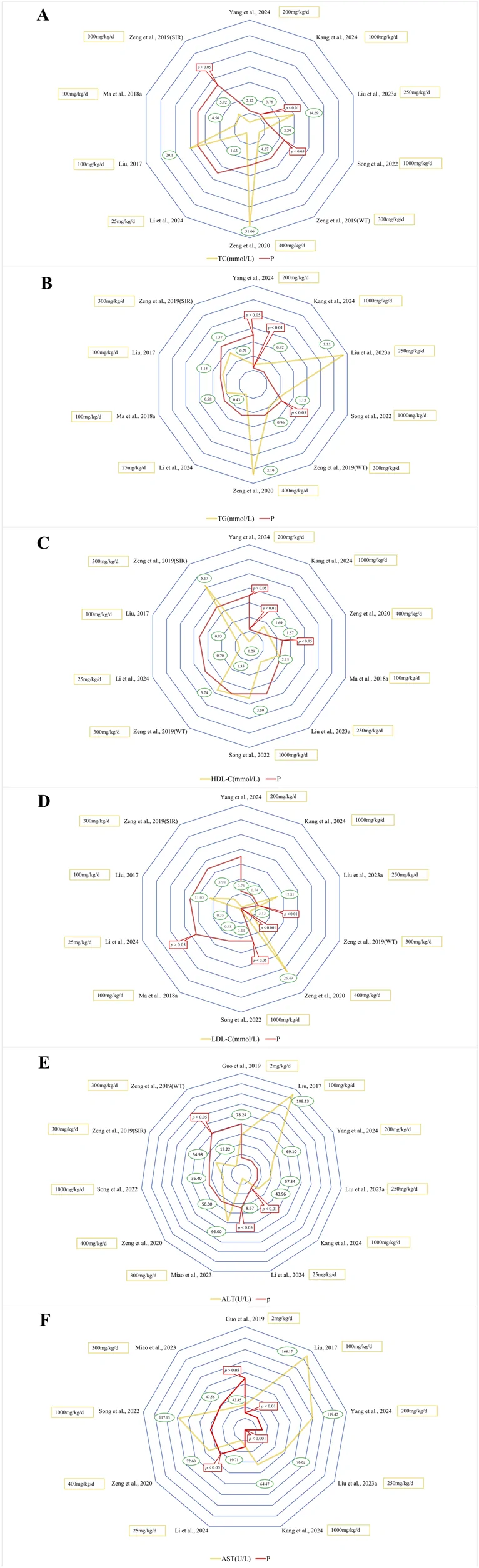
Dose-response radar plots of the effects of DHM treatment on **(A)** serum TC, **(B)** serum TG, **(C)** HDL-C, **(D)** LDL-C, **(E)** ALT, and **(F)** AST.

Exploratory dose-stratified subgroup analysis showed that the pooled mean MD in the high-dose subgroup (≥100 mg/kg/day) was −2.18 (95% CI –3.17 to −1.19). The low-dose (≤100 mg/kg/day) stratum was represented by a single study, which individually reported an MD of −0.64 (95% CI –0.79 to −0.49). Accordingly, no pooled estimate was computed for this stratum, and a formal between-subgroup comparison was not conducted. Duration-stratified subgroup analysis did not reveal a significant between-subgroup difference (*p* = 0.58, I^2^ = 0%). The pooled effect estimate was −1.63 (95% CI –2.76 to −0.50) for short-term interventions (≤8 weeks) and −2.08 (95% CI –3.20 to −0.96) for long-term interventions (≥8 weeks) (Figures 10A, 11A). Taken together, the 3D visualization and radar plot suggested that higher doses (≥100 mg/kg/day) may be associated with greater reductions in TC. However, the apparent advantage observed within the 8–12-week treatment interval was not supported by formal subgroup testing (Figures 12A, 13A).

#### Serum TG

Dose-stratified subgroup analysis indicated that the pooled MD in the high-dose subgroup (≥100 mg/kg/day) was −0.41 (95% CI –0.71 to −0.12). With only one study in the low-dose range (≤100 mg/kg/day), the reported MD of −0.79 (95% CI –0.96 to −0.62). Subgroup analysis by treatment duration did not show a significant between-group difference (*p* = 0.90, I^2^ = 0%). The pooled reduction in TG was −0.45 (95% CI –0.79 to −0.10) for short-term (≤8 weeks) interventions and −0.48 (95% CI –0.84 to −0.12) for long-term (≥8 weeks) interventions (Figures 10B, 11B). Findings from the 3D visualization and radar plot suggested a possible trend toward greater TG reduction at doses ≥100 mg/kg/day and with longer treatment durations (Figures 12B, 13B).

#### HDL-C

The pooled MD in the high-dose subgroup (≥100 mg/kg/day) was 0.48 (95% CI 0.07 to 0.90). The low-dose stratum (≤100 mg/kg/day) was represented by a single study, which individually reported an MD of 0.36 (95% CI 0.21 to 0.51). Duration-stratified analysis revealed no significant between-subgroup difference (p = 0.71, I^2^ = 0%). The pooled effect estimate was 0.39 (95% CI 0.26 to 0.52) for short-term (≤8 weeks) interventions and 0.49 (95% CI −0.01 to 0.99) for long-term (≥8 weeks) interventions. Notably, the effect observed in the long-term subgroup did not reach statistical significance (*p* = 0.06) (Figures 10C, 11C). Consistent with these findings, the 3D visualization and radar plot did not suggest a clear dose- or duration-dependent gradient in HDL-C improvement. Therefore, the available evidence does not permit firm conclusions regarding dose-dependent effects or treatment duration–related trends in HDL-C (Figures 12C, 13C).

#### LDL-C

The pooled MD in the high-dose subgroup (≥100 mg/kg/day) was −0.70 (95% CI –1.05 to −0.34). Only one study fell into the low-dose stratum (≤100 mg/kg/day), with an individually reported MD of −0.09 (95% CI –0.13 to −0.05). Duration-stratified analysis identified a significant difference between treatment-duration categories (*p* = 0.002, I^2^ = 90%). The pooled MD was −0.23 (95% CI –0.45 to −0.02) for short-term (≤8 weeks) interventions and −1.56 (95% CI –2.35 to −0.77) for long-term (≥8 weeks) interventions (Figures 10D, 11D). Taken together, the 3D visualization and radar plot suggested that greater reductions in LDL-C may be associated with doses ≥100 mg/kg/day administered for at least 8 weeks (Figures 12D, 13D).

#### ALT

The pooled MD in the high-dose subgroup (≥100 mg/kg/day) was −30.15 (95% CI –39.87 to −20.43). Only one study contributed to the low-dose stratum (≤100 mg/kg/day), yielding an individual MD of −9.51 (95% CI –11.49 to −7.53). Duration-stratified analysis identified a significant subgroup effect (*p* = 0.01, I^2^ = 83.3%). The pooled reduction in ALT was −13.89 (95% CI –21.32 to −6.46) for short-term (≤8 weeks) interventions and −36.91 (95% CI –53.79 to −20.03) for long-term (≥8 weeks) interventions (Figures 10E, 11E). Inspection of the 3D visualization and radar plot may suggest a potential trend toward ALT reductions at longer treatment durations (Figures 12E, 13E).

#### AST

The pooled MD in the high-dose subgroup (≥100 mg/kg/day) was −44.92 (95% CI –67.76 to −22.09). The low-dose stratum (≤100 mg/kg/day) was informed by only one investigation, which reported an MD of −14.00 (95% CI –18.62 to −9.38). Duration-stratified analysis did not reveal a significant between-subgroup difference (*p* = 0.06, I^2^ = 71.6%). The pooled reduction in AST was −14.07 (95% CI –16.96 to −11.19) for short-term (≤8 weeks) interventions and −59.34 (95% CI –106.50 to −12.18) for long-term (≥8 weeks) interventions (Figures 10F, 11F). The 3D visualization and radar plot further suggest that AST may decrease more substantially with longer treatment duration, whereas the dose-response surface within the tested range appears relatively flat (Figures 12F, 13F).

### Sensitivity analysis

Sensitivity analyses were conducted for serum TC, serum TG, HDL-C, LDL-C, and ALT levels. Sequential exclusion of individual studies did not substantially alter the pooled effect sizes, indicating that the overall findings were robust and reliable.

### Publication bias

**FIGURE 14:**
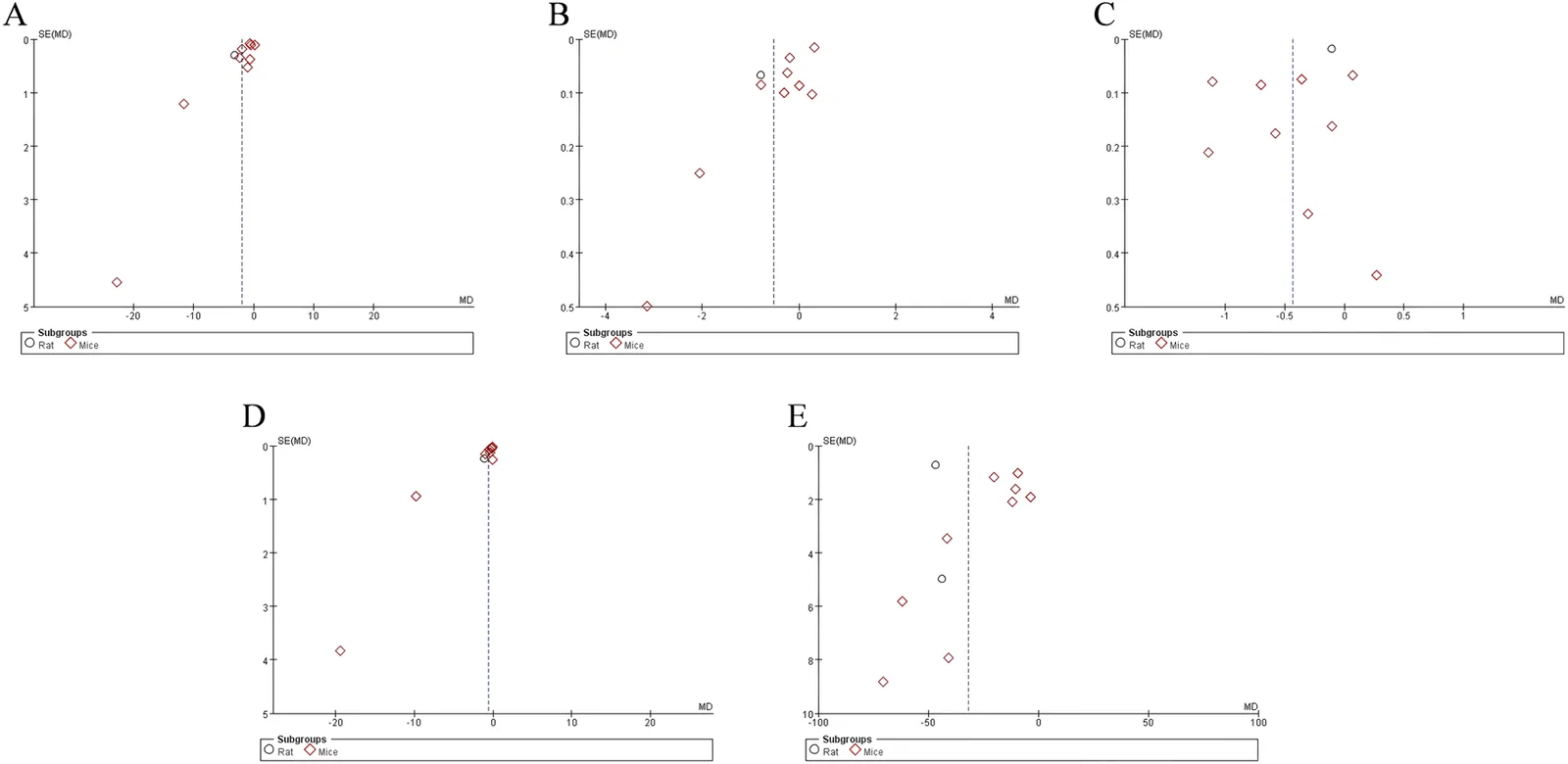
Publication bias assessment for **(A)** serum TC, **(B)** serum TG, **(C)** HDL-C, **(D)** LDL-C, and **(E)** ALT.

Figure 14 shows the publication bias assessment for serum TC, serum TG, HDL-C, LDL-C, and ALT. Funnel plots reveal that most studies on serum TC, serum TG, and LDL-C are concentrated in the upper part of the plots, indicating high precision. However, the observed asymmetry suggests potential publication bias or small-study effects, as smaller studies with less favorable results may remain unpublished. The plots also indicate significant heterogeneity across all outcomes (serum TC, serum TG, HDL-C, LDL-C, and ALT), which may be due to variations in animal models, DHM doses, study designs, or measurement methods. The limited number of studies included in the meta-analysis may also contribute to this variability.

Due to the limited number of studies (<10) for most outcomes, publication bias was not formally assessed. However, the funnel plot asymmetry suggests that publication bias may affect the results. Future studies with larger sample sizes and statistical tests are needed to confirm these findings and provide more reliable estimates of DHM’s pharmacological effects in treating NAFLD.

## Discussion

Vine tea is a traditional Chinese medicinal tea with a long history of use and widespread application. From a botanical standardization perspective, approximately 57 metabolites have been reported in vine tea to date, including flavonoids, phenolic acids, steroids, and volatile metabolites (Wu et al., 2023). Among them, DHM is the most abundant flavonoid, and its content in dried leaves can reach up to 30% of the dry weight (Hu et al., 2020; Yu et al., 2021). This DHM-dominated chemical profile provides favorable conditions for the standardization of plant-derived preparations. Currently, analytical methods such as HPLC, HPLC-MS, and HPLC-PDA have been employed for the quantitative determination of DHM and related flavonoids (Zeng et al., 2023), thereby improving the consistency of quality control for raw materials and preparations and offering technical support for subsequent pharmacological research and formulation development. However, the variety source of vine tea has yet to be standardized. Significant differences in the contents of total flavonoids and DHM have been observed among samples from different geographical origins and plant parts, including buds, young leaves, mature leaves, and tea stalks (Xie et al., 2019; Li et al., 2020). This standardization issue may substantially affect the bioequivalence of DHM preparations and reproducibility across studies, and represents a critical quality-control aspect that warrants particular attention in future preclinical and clinical investigations of DHM.

In addition to preclinical investigations, a small number of randomized, double-blind, placebo-controlled clinical trials involving DHM-based preparations have been reported in recent years (Wang et al., 2025d; Chen et al., 2015). These studies indicate that intervention with DHM-based preparations can improve certain liver function parameters, indices of glucose and lipid metabolism, and liver-related outcomes in patients with NAFLD. These preliminary clinical observations are broadly consistent with improvements in hepatic lipid accumulation, dyslipidemia, liver function abnormalities, and glucose metabolism dysregulation observed in the included animal studies. Notably, the subgroup analysis and dose–time effect analysis conducted herein suggest that treatment duration may influence pharmacological effects, and the benefits observed in existing clinical studies have predominantly been reported after longer intervention periods (Michailidou et al., 2026). This suggests that sustained exposure may contribute to the metabolic regulatory effects of DHM.

However, the number of relevant clinical studies remains limited, the sample sizes are relatively small, and the formulations, doses, and endpoints employed vary across studies, thus precluding the establishment of a definitive clinical application regimen at present. Furthermore, the translational relevance of DHM-containing preparations still faces several challenges (Ren et al., 2026; Zhang et al., 2022b). Studies have shown that its oral bioavailability is low (Ye et al., 2021), which may be attributed to limited aqueous solubility and intestinal permeability, potentially affecting its *in vivo* exposure levels and the consistency of therapeutic responses (Li et al., 2024a; Liu et al., 2019). Therefore, future research should strengthen pharmacokinetic evaluation, formulation optimization, and the conduct of higher-quality clinical trials to further clarify the optimal dosing strategy and long-term benefits of DHM.

### Summary of evidence

This systematic review and meta-analysis evaluated the pharmacological effects of DHM across preclinical NAFLD models and examined whether treatment dose, duration, and model type influenced these effects. Across the included studies, DHM was generally associated with improvements in lipid-related parameters, liver injury markers, and glucose-related parameters. However, the magnitude of these effects differed among experimental models, suggesting that disease characteristics and model-specific mechanisms may influence responses to DHM treatment. Subgroup analyses suggested that higher doses (≥100 mg/kg/day) were associated with larger reductions in TC, LDL-C, ALT, and AST, while interventions lasting at least 8 weeks tended to show greater improvements in LDL-C and AST. In contrast, no consistent relationship between dose or treatment duration and changes in TG or HDL-C was observed. Interpretation of these findings requires caution because several subgroups comprised only a single study, and considerable heterogeneity persisted in many analyses. Accordingly, the subgroup results should be regarded as exploratory rather than confirmatory. These findings suggest that differences in dose, duration, and model type may contribute to variation in treatment effects but are unlikely to fully explain the observed heterogeneity. Taken together, the current evidence suggests a pharmacological potential of DHM in preclinical NAFLD, although further studies using standardized experimental designs are needed to clarify factors influencing treatment response and to better define intervention strategies.

### Potential protective mechanisms of DHM in NAFLD

The pharmacological effects of DHM in preclinical NAFLD models involve several interconnected processes (Weidmann, 2012; Zai et al., 2018), including attenuation of oxidative stress (Ding et al., 2018), suppression of inflammatory signaling, and modulation of lipid (Yu et al., 2022) and glucose metabolism (Cobbina and Akhlaghi, 2017; Kumar et al., 2021; Wei et al., 2025). The subgroup patterns observed in this analysis, particularly the outcome-specific differences in dose- and duration-dependence, suggest that the relative contribution of these mechanisms may vary across different dose-duration conditions. Nevertheless, given the exploratory nature of these subgroup findings and the considerable heterogeneity across studies, this interpretation should be considered hypothesis-generating rather than conclusive, and warrants further investigation in well-designed preclinical studies.

Oxidative stress is a key driver of NAFLD progression from simple steatosis to steatohepatitis (Ma et al., 2021). Studies have shown that DHM increases superoxide dismutase and catalase activities while restoring glutathione levels and reducing malondialdehyde (MDA) concentrations (Zhang et al., 2022a). Activation of the Nrf2/HO-1 pathway is considered a major mechanism through which DHM enhances endogenous antioxidant defenses and limits ROS accumulation (Liu et al., 2022). Chronic low-grade inflammation is another pathogenic hallmark of NAFLD (Ortiz-López et al., 2022). DHM reduces pro-inflammatory mediators such as TNF-α, IL-6, and IL-1β, and suppresses NF-κB and MAPK cascades (Wei et al., 2024; Zhou et al., 2022; Zhao et al., 2021). At the same time, DHM enhances insulin sensitivity by improving phosphorylation of insulin receptor substrate-1 and activating the PI3K/Akt pathway (Babotă et al., 2024). Regarding lipid metabolism, DHM activates AMPK while inhibiting SREBP-1c and fatty acid synthase (González-Garibay et al., 2025), thereby curbing *de novo* lipogenesis and favoring fatty acid utilization (Gong et al., 2022; Chen et al., 2018).

Emerging evidence also implicates the gut–liver axis in the hepatoprotective effects of DHM(Canivet et al., 2021; Yang et al., 2024b). This metabolite alters intestinal microbial composition (Kang et al., 2024; Song et al., 2022), increasing the relative abundance of *Lactobacillus* and Bacteroidetes while reducing potentially pathogenic taxa (Hu et al., 2024). It further reinforces the intestinal barrier by upregulating tight junction proteins (Wang et al., 2024), thereby limiting the translocation of gut-derived endotoxins into the portal circulation.

Subgroup analyses suggested that higher DHM doses were associated with greater reductions in TC and LDL-C. This observation is consistent with experimental evidence showing that DHM activates AMPK and suppresses SREBP-1c-mediated lipogenesis. In contrast, the greater improvement in AST observed with longer treatment durations may reflect the gradual attenuation of hepatocellular injury and inflammatory responses, potentially involving the cumulative effects of NF-κB suppression over time. Nevertheless, because the included studies did not systematically evaluate mechanistic changes across different dose levels or treatment durations, these interpretations remain speculative and should be validated in future mechanistic studies investigating dose-response and time-course relationships.

### DHM in the context of other natural hepatoprotective metabolites

In the field of flavonoid research, quercetin and resveratrol are the two most widely studied reference metabolites (Shen et al., 2022; Solnier et al., 2023; Zhang et al., 2024). Quercetin exerts its effects primarily through antioxidant and anti-inflammatory mechanisms (Qi et al., 2022), but evidence regarding its role in the regulation of energy metabolism and the regulation of autophagy is limited (Alizadeh and Ebrahimzadeh, 2022). Resveratrol improves metabolic homeostasis via the SIRT1 pathway (Lagouge et al., 2006), but its effects are inconsistent across different NAFLD models (Yang et al., 2022). Among other natural hepatoprotective metabolites investigated in NAFLD (Ebrahimzadeh et al., 2024), silymarin has been widely studied for its antioxidant and membrane-stabilizing properties. However, variability in bioavailability and metabolic responses has been reported, which may contribute to differences in treatment outcomes across studies (Li et al., 2024b; Wang et al., 2025a). Curcumin has also attracted considerable attention because of its anti-inflammatory and lipid-lowering activities. Nevertheless, its relatively low systemic bioavailability and rapid metabolism remain important considerations when interpreting findings from preclinical and clinical investigations (Han et al., 2024; Li et al., 2024c).

Evidence from the studies included in this review suggests that DHM may modulate several interconnected biological processes, including autophagy, mitochondrial homeostasis, and gut–liver axis function. In metabolic regulation, DHM not only activates the AMPK–PGC-1α–PPARα pathway to promote β-oxidation (Lei et al., 2020; Zhang et al., 2018b), but also enhances lysosomal function and selective autophagy, reducing lipid droplet accumulation at its source (Zai et al., 2018). In mitochondrial homeostasis, DHM simultaneously modulates the SIRT1/SIRT3 pathway, improving mitochondrial respiratory chain function and redox balance (Zeng et al., 2024). At the gut–liver axis level, DHM reshapes the gut microbiota, strengthens intestinal barrier function (Wang et al., 2025b), and reduces endotoxin load (Lei et al., 2020). In addition, previous animal studies have also reported favorable pharmacokinetic and safety characteristics of DHM. Collectively, these observations support further exploration of the pharmacological potential of DHM for NAFLD in preclinical settings, although its translational relevance remains to be established in future clinical studies.

However, this comparison represents a narrative synthesis based on the reviewed DHM studies and the available literature on other hepatoprotective metabolites. Because these observations are based on indirect comparisons across studies with different animal models, experimental conditions, outcome measures, and study designs, no definitive conclusions about the relative pharmacological effects of DHM can be drawn. Direct head-to-head comparative studies are needed to further evaluate these potential differences.

### Safety considerations and toxicological evidence

The doses of DHM used in preclinical NAFLD models (25–1,000 mg/kg/day) translate, based on interspecies scaling, into relatively high human-equivalent doses. Taking the commonly used dose of 100 mg/kg/day in rodent models as an example, and applying the FDA-recommended allometric scaling approach (Km factor: mouse = 3, human = 37), the estimated human equivalent dose is approximately 8.1 mg/kg/day, which corresponds to approximately 486 mg/day for a 60-kg adult. Although such exposure levels are unlikely to be achieved through conventional dietary intake alone, they may be achievable through standardized DHM-enriched botanical extracts or formulation approaches designed to enhance bioavailability (Saikia et al., 2026). Applying a conventional 10-fold safety factor yields a theoretical conservative starting dose for first-in-human studies could be estimated at approximately 0.81 mg/kg/day (about 50 mg/day for a 60-kg adult), which may provide a reference point for future dose selection in first-in-human studies. Any subsequent dose escalation toward the projected therapeutic range would require appropriate pharmacokinetic characterization and safety monitoring.

Although DHM is a naturally occurring flavonoid with a history of dietary exposure, pharmacokinetic profiling and dose-optimization studies are required to determine clinically achievable and effective dosing strategies. Safety evaluation is a critical prerequisite for the long-term clinical application of DHM, particularly given the chronic nature of NAFLD and the high doses employed in animal studies. Available preclinical evidence suggests that DHM exhibits low acute toxicity and is generally well tolerated in rodents, even at relatively high doses, with no overt adverse effects on liver or kidney function reported in short-term studies (Chien et al., 2019; Qi et al., 2024a). However, comprehensive data regarding long-term administration, reproductive toxicity, and potential drug–drug interactions remain limited. Importantly, chronic metabolic diseases such as NAFLD require sustained therapeutic intervention, underscoring the necessity of rigorous subchronic and chronic toxicity assessments. Accordingly, further toxicological investigations and well-designed clinical safety studies are essential before DHM can be considered a viable therapeutic candidate for long-term use in patients with NAFLD.

### Limitations and future directions

This analysis has several shortcomings. Only Chinese and English databases were queried, which may introduce selection bias. The preponderance of positive reports raises concern for publication bias. Most included works provided limited information on randomization, blinding, and allocation concealment. Although subgroup comparisons were conducted by dose and treatment duration, heterogeneity remained high within strata. Residual variability likely stems from divergent experimental designs, assay methods, or technical execution across laboratories. Inadequate reporting of the characteristics of DHM preparations may affect the reproducibility of research findings and could be a potential source of heterogeneity across studies. Since the subgroup cutoffs of 100 mg/kg/day and 8 weeks were not prespecified in the study protocol but were determined retrospectively based on study distribution, the results of the corresponding subgroup analyses should be considered exploratory and require further validation in future studies. Finally, current animal models do not fully recapitulate the intricate pathophysiology of human NAFLD, warranting caution when extrapolating preclinical observations to clinical settings. Incomplete methodological reporting in primary studies limits the inferences that pooled analyses can support (Robinson and Liu, 2025).

It is recommended that future preclinical studies report DHM-related characteristics more comprehensively, including source, purity, extraction method, and formulation stability, to enhance the transparency, reproducibility, and comparability of research results. Such studies should be designed to evaluate mechanistic outcomes across different dose ranges and treatment durations, which may help clarify the biological basis of the observed dose-related and time-related effects. Future preclinical studies may benefit from improved methodological standardization and reporting completeness to enhance the robustness and reproducibility of the evidence base. More detailed reporting of NAFLD induction protocols, including dietary composition and treatment regimens, could improve cross-study comparability. Harmonization of outcome assessment time points and adoption of a core set of biochemical and histopathological endpoints may further facilitate consistent interpretation and data synthesis. Inclusion of both male and female animals may help clarify potential sex-dependent effects of DHM. In addition, strengthened adherence to ARRIVE guidelines (Percie du Sert et al., 2020), particularly regarding randomization, blinding, and sample size estimation, may further improve transparency and reproducibility.

The findings of the present study may provide preliminary guidance for the future clinical development of DHM. Based on the available evidence, a stepwise translational research strategy may be considered in future investigations. Dose selection in early-phase clinical studies may be informed, in part, by human-equivalent dose estimates derived from preclinical evidence, while appropriate safety monitoring may facilitate the gradual exploration of suitable dosing regimens. The observed association between higher DHM doses and greater improvements in lipid-related parameters, together with the more pronounced effects of longer treatment durations on liver injury markers, suggests that both dose level and treatment duration may warrant consideration during the design of future translational and clinical studies. Lipid-related parameters, such as TC and LDL-C, may serve as potential early pharmacodynamic indicators, whereas liver function markers and imaging- or histology-based outcomes may provide insights into longer-term pharmacological responses. Given the currently limited clinical evidence for DHM, future studies may also benefit from incorporating pharmacokinetic assessments, formulation optimization strategies, and dose-ranging investigations to enhance translational applicability. Collectively, these considerations provide a preliminary framework for future clinical research evaluating the pharmacological effect and safety of DHM in patients with NAFLD, although further validation through well-designed clinical studies remains necessary.

## Conclusion

This systematic review and meta-analysis suggest that DHM treatment may improve lipid metabolism and liver injury markers in preclinical NAFLD models. Exploratory stratified analyses revealed outcome-specific trends, with lipid parameters appearing to be more responsive to dose and hepatic enzyme responses potentially more closely related to treatment duration. However, substantial heterogeneity and incomplete methodological reporting limit the strength of available evidence. Future studies may benefit from more standardized experimental designs and reporting practices, including pharmacokinetic-informed dose-ranging strategies, harmonized reporting, and the inclusion of both male and female animals, to improve reproducibility and translational relevance.

## Data Availability

The original contributions presented in the study are included in the article/Supplementary Material, further inquiries can be directed to the corresponding authors.
