# Supplementary material for: Pharmacological effects of dihydromyricetin in preclinical models of NAFLD: a systematic review and meta-analysis stratified by dose and duration
Source: Front Pharmacol. 2026 Aug 6;17:1863189. doi: 10.3389/fphar.2026.1863189 (PMC13490083; doi:10.3389/fphar.2026.1863189)
Supplement: Supplementary file 1 [file Supplementaryfile1.docx]

**Supplementary Material 1: search strategy**

1.Search strategies of PubMed (Searched from inception to Aug. 01, 2025 and found 35 literature)

Table1

PubMed search strategy.

| PubMed search strategy | |
| --- | --- |
| #1. | Non alcoholic Fatty Liver Disease [mh] |
| #2. | Fatty Liver, Nonalcoholic [mh] |
| #3. | Fatty Livers, Nonalcoholic [mh] |
| #4. | Liver, Nonalcoholic Fatty [mh] |
| #5. | Livers, Nonalcoholic Fatty [mh] |
| #6. | Nonalcoholic Fatty Liver [mh] |
| #7. | Nonalcoholic Fatty Livers [mh] |
| #8. | NAFLD [mh] |
| #9. | Nonalcoholic Fatty Liver Disease [mh] |
| #10. | Nonalcoholic Steatohepatitis [mh] |
| #11. | Nonalcoholic Steatohepatitides [mh] |
| #12. | Steatohepatitides, Nonalcoholic [mh] |
| #13. | Steatohepatitis, Nonalcoholic [mh] |
| #14. | #1 OR #2 OR #3 OR #4 OR #5 OR #6 OR #7 OR #8 OR #9 OR #10 OR #11 OR #12 OR #13 |
| #15. | dihydromyricetin |
| #16. | #14 AND #15 |
| #17. | Animals [all] not humans [all] |
| #18. | Rat. [all] |
| #19. | Mice [all] |
| #20. | Experiment [all] |
| #21. | #17 OR #18 OR #19 OR #20 |
| #22. | #16 AND #21 |

2.Search strategies of EMBASE (Searched from inception to Aug. 01, 2025 and found 64 literature)

Table 2

EMBASE search strategy.

| EMBASE search strategy | |
| --- | --- |
| #1. | nonalcoholic fatty liver.mp. |
| #2. | NAFLD (nonalcoholic fatty liver disease)/ |
| #3. | non alcoholic fatty liver disease/ |
| #4. | non alcoholic hepato-steatosis/ |
| #5. | non alcoholic hepatosteatosis/ |
| #6. | non alcoholic liver steatosis/ |
| #7. | non alcoholic steatotic hepatopathy/ |
| #8. | non-alcoholic fatty liver/ |
| #9. | non-alcoholic fatty liver disease/ |
| #10. | non-alcoholic FLD/ |
| #11. | non-alcoholic hepatic steatosis/ |
| #12. | nonalcoholic fatty liver disease/ |
| #13. | nonalcoholic FLD/ |
| #14. | nonalcoholic hepatic steatosis/ |
| #15. | nonalcoholic hepatosteatosis/ |
| #16. | nonalcoholic liver steatosis/ |
| #17. | nonalcoholic fatty liver/ |
| #18. | #1 OR #2 OR #3 OR #4 OR #5 OR #6 OR #7 OR #8 OR #9 OR #10 OR #11 OR #12 OR #13 OR #14 OR #15 OR #16 OR #17 |
| #19. | dihydromyricetin |
| #20. | #18 AND #19 |
| #21. | experimental animal.mp. |
| #22. | experimental animals/ |
| #23. | experimental animal/ |
| #24. | laboratory animal/ |
| #25. | human.mp |
| #26. | #21 OR #22 OR #23 OR #24 NOT #25 |
| #27. | rat.mp |
| #28. | rats/ |
| #29. | rat/ |
| #30. | #27 OR #28 OR #29 |
| #31. | mouse.mp |
| #32. | mice/ |
| #33. | mouse/ |
| #34. | #31 OR #32 OR #33 |
| #35. | #26 OR #30 OR #34 |
| #36. | #18 AND #19 AND #35 |

3.Search strategies of Web of Science (Searched from inception to Aug. 01, 2025 and found 84 literature)

Table 3

Web of Science search strategy.

| Web of Science search strategy | |
| --- | --- |
| #1. | Non alcoholic Fatty Liver Disease [mh] |
| #2. | Fatty Liver, Nonalcoholic [mh] |
| #3. | Fatty Livers, Nonalcoholic [mh] |
| #4. | Liver, Nonalcoholic Fatty [mh] |
| #5. | Livers, Nonalcoholic Fatty [mh] |
| #6. | Nonalcoholic Fatty Liver [mh] |
| #7. | Nonalcoholic Fatty Livers [mh] |
| #8. | NAFLD [mh] |
| #9. | Nonalcoholic Fatty Liver Disease [mh] |
| #10. | Nonalcoholic Steatohepatitis [mh] |
| #11. | Nonalcoholic Steatohepatitides [mh] |
| #12. | Steatohepatitides, Nonalcoholic [mh] |
| #13. | Steatohepatitis, Nonalcoholic [mh] |
| #14. | #1 OR #2 OR #3 OR #4 OR #5 OR #6 OR #7 OR #8 OR #9 OR #10 OR #11 OR #12 OR #13 |
| #15. | dihydromyricetin |
| #16. | #14 AND #15 |
| #17. | Animals [all] not humans [all] |
| #18. | Rat. [all] |
| #19. | Mice [all] |
| #20. | Experiment [all] |
| #21. | #17 OR #18 OR #19 OR #20 |
| #22. | #16 AND #21 |

4.Search strategies of Cochrane Central Register of Controlled Trials (CENTRAL) (Searched from inception to Aug. 01, 2025 and found 1 literature)

Cochrane search strategy:

| #1 | (Non alcoholic Fatty Liver Disease):ti,ab,kw OR (Fatty Liver, Nonalcoholic):ti,ab,kw OR (Fatty Livers, Nonalcoholic):ti,ab,kw OR (Liver, Nonalcoholic Fatty):ti,ab,kw kw OR (Nonalcoholic Fatty Liver):ti,ab,kw OR (Nonalcoholic Fatty Livers):ti,ab,kw OR (NAFLD):ti,ab,kw OR (Nonalcoholic Fatty Liver Disease):ti,ab,kw OR (Nonalcoholic Steatohepatitis):ti,ab,kw OR (Nonalcoholic Steatohepatitides):ti,ab,kw OR (Steatohepatitides, Nonalcoholic):ti,ab,kw OR (Steatohepatitis, Nonalcoholic):ti,ab,kw |
| --- | --- |
| #2 | (dihydromyricetin):ti,ab,kw |
| #3 | #1 AND #2 |
| #4 | (Animals):ti,ab,kw |
| #5 | #3 AND #4 |

5.Search strategies of CNKI (Searched from inception to Aug. 01, 2025 and found 15 literature)

CNKI search strategy:

非酒精性脂肪肝AND 二氢杨梅素 AND 实验

6.Search strategies of Wanfang (Searched from inception to Aug. 01, 2025 and found 22 literature)

Wanfang Database search strategy:

非酒精性脂肪肝AND 二氢杨梅素 AND 实验

7.Search strategies of VIP (Searched from inception to Aug. 01, 2025 and found 16 literature)

VIP search strategy:

非酒精性脂肪肝AND 二氢杨梅素 AND 实验
